# The nucleolar protein SAHY1 is involved in pre-rRNA processing and normal plant growth

**DOI:** 10.1093/plphys/kiaa085

**Published:** 2020-12-29

**Authors:** Pei-jung Hsu, Mei-Chen Tan, Hwei-Ling Shen, Ya-Huei Chen, Ya-Ying Wang, San-Gwang Hwang, Ming-Hau Chiang, Quang-Vuong Le, Wen-Shuo Kuo, Ying-Chan Chou, Shih-Yun Lin, Guang-Yuh Jauh, Wan-Hsing Cheng

**Affiliations:** 1 Institute of Plant and Microbial Biology, Academia Sinica, Taipei, Taiwan; 2 Graduate Institute of Life Sciences, National Defense Medical Center, Taipei, Taiwan; 3 Department of Biological Science and Technology, National Pingtung University of Science and Technology, Neipu, Pingtung County,Taiwan

## Abstract

Although the nucleolus is involved in ribosome biogenesis, the functions of numerous nucleolus-localized proteins remain unclear. In this study, we genetically isolated *Arabidopsis thaliana salt hypersensitive mutant 1* (*sahy1*), which exhibits slow growth, short roots, pointed leaves, and sterility. *SAHY1* encodes an uncharacterized protein that is predominantly expressed in root tips, early developing seeds, and mature pollen grains and is mainly restricted to the nucleolus. Dysfunction of SAHY1 primarily causes the accumulation of 32S, 18S-A_3_, and 27SB pre-rRNA intermediates. Coimmunoprecipitation experiments further revealed the interaction of SAHY1 with ribosome proteins and ribosome biogenesis factors. Moreover, *sahy1* mutants are less sensitive to protein translation inhibitors and show altered expression of structural constituents of ribosomal genes and ribosome subunit profiles, reflecting the involvement of SAHY1 in ribosome composition and ribosome biogenesis. Analyses of ploidy, S-phase cell cycle progression, and auxin transport and signaling indicated the impairment of mitotic activity, translation of auxin transport carrier proteins, and expression of the auxin-responsive marker DR5::GFP in the root tips or embryos of *sahy1* plants. Collectively, these data demonstrate that SAHY1, a nucleolar protein involved in ribosome biogenesis, plays critical roles in normal plant growth in association with auxin transport and signaling.

## Teaser

SALT-HYPERSENSITIVE MUTANT 1, a nucleolar protein involved in ribosome biogenesis, regulates the auxin-mediated development of vegetative and reproductive tissues.

## Introduction

The nucleolus is the largest subnuclear compartment and plays important roles in ribosome biogenesis (Boisvert et al., 2007; Shaw and Brown, 2012; Weis et al., 2015). Eukaryotic ribosomes are composed of two subunits: the 60S large subunit consists of three rRNAs (5S, 5.8S, and 25S–28S) and approximately 47 different ribosomal proteins (RPs); the 40S small subunit (SSU) consists of a single 18S rRNA and 33 different RPs (Wilson and Doudna Cate, 2012). In Arabidopsis, these 80 RPs have largely been confirmed by proteomic analyses of cytosolic ribosomes or proteins in the nucleoplasm and the nucleolus (Chang et al., 2005; Pendle et al., 2005; Carroll et al., 2008; Palm et al., 2016; Montacié et al., 2017). Functional 80S ribosomes are assembled from a combination of the large and small ribosome subunits and are responsible for protein translation by association with messenger RNA in the cytosol. In plants, ribosomes are highly heterogeneous because, as shown in *Arabidopsis*, each RP is part of a family of multiple members that might be differentially expressed at different developmental stages, in different tissues or cell types, or in response to different environmental stimuli (Barakat et al., 2001; Hummel et al., 2012; Shaw and Brown, 2012; Carroll, 2013).

The biogenesis of eukaryotic ribosomes, including the transcription of rDNA, processing, and modification of precursor rRNAs (pre-rRNAs), and assembly of RPs, is a fundamental cellular process that involves hundreds of ribosome biogenesis factors (RBFs). Pre-rRNA processing and ribosome biogenesis are complex and highly coordinated processes that have been intensively studied in yeast (Fatica and Tollervey, 2002). The steps of ribosome biogenesis that are generally conserved are expected to coexist in metazoans and plants (Brown and Shaw, 1998; Carroll et al., 2008; Weis et al., 2015; Sáez-Vasquez and Delseny, 2019). In general, ribosome biogenesis initially begins with the transcription of tandem repeat rDNA by RNA polymerase I (Pol I; Pontvianne et al., 2013) to form the 45S pre-rRNA in the nucleolus. This long primary pre-rRNA contains three rRNA coding sequences (18S, 5.8S, 25–28S) that are separated by two internal transcribed spacers (ITSs; ITS1 and ITS2) and flanked by external transcribed spacers (ETSs; 5′-ETS and 3′-ETS). However, the 5S rRNA is separately transcribed from the other rDNA by Pol III (Layat et al., 2012) in the nucleus. After it is cleaved, the 45S pre-rRNA is converted to a 35S pre-rRNA intermediate (Sáez-Vasquez et al., 2004; Comella et al., 2008; Zakrzewska-Placzek et al., 2010) that subsequently follows one of two pathways. Pathway 1, also known as the 5′-ETS-first pathway, is the primary pathway in yeast, in which the 5′-ETS is removed before cleavage within ITS1 (Hang et al., 2014; Fernández-Pevida et al., 2015). In contrast, pathway 2, the ITS1-first pathway, involves the cleavage of ITS1 prior to 5′-ETS cleavage, which is common in metazoans for the production of mature rRNA. The two pathways may coexist in plants (Hang et al., 2014; Weis et al., 2015; Tomechi et al., 2017), which is reminiscent of the strict processing pathways found in yeast. Recently, an ITS2-first cleavage pathway involved in the maturation of the plant-specific precursor 5′-5.8S during plant development was identified (Palm et al., 2019).

In yeast, approximately 250 RBFs of several protein classes are involved in ribosome biogenesis (Woolford and Baserga, 2013). More RBFs are expected to exist in higher plants than in yeast because of the complex ribosome biogenesis process in the former. Some genes involved in ribosome biogenesis in plants do not exist in yeast, and some RBFs of yeast have no homologous genes in plants, suggesting that plants might have specific RBFs that function differently from those of yeast (Simm et al., 2015; Weis et al., 2015; Palm et al., 2019), presumably due to long-term evolution. With respect to the importance of pre-rRNA processing and ribosome biogenesis in humans, genetic evidence indicates that mutations in RPs or RBFs lead to aberrant pre-rRNA processing and polyadenylation-mediated control, resulting in severe disease (Narla and Ebert, 2010; McCann and Baserga, 2013). Similarly, different RPs or RBFs in plants may contribute differently to the development of vegetative or reproductive tissues (Horiguchi et al., 2011). Thus, the dysfunction of ribosome biogenesis-related genes may cause defects in gametogenesis, embryogenesis, or normal plant growth and development (Byrne, 2009; Carroll, 2013; Weis et al., 2015).

Although numerous RBFs participate in pre-rRNA processing and ribosome biogenesis/assembly in plants, very few of these genes have been characterized to date. In this study, a genetic screening was conducted to identify *salt-hypersensitive mutant 1* (*sahy1*) plants, which displayed slow growth and improper vegetative and reproductive development. The mutation of *SAHY1* caused defects in pre-rRNA processing and altered both the ribosome profile and the expression of RP and RBF genes. A coimmunoprecipitation (Co-IP) study further revealed that SAHY1 was associated with both RPs and RBFs. Thus, SAHY1 plays important roles in ribosome biogenesis and normal plant growth.

## Results

### The mutation of *SAHY1* alters salt sensitivity and plant growth

**Figure 1:**
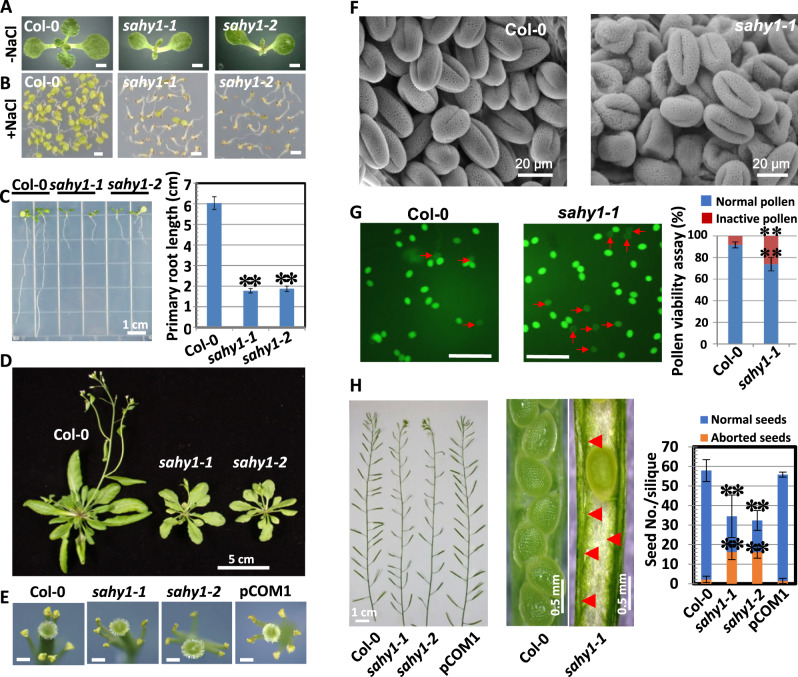
Mutation of Arabidopsis *SAHY1* alters salt sensitivity and plant growth. A, B, Comparison of seedlings under normal or salt-stressed growth conditions. Seedlings were grown on basal medium (A) or medium containing 150-mM NaCl (B) for 10 d. Scale bars = 1 mm in (A) and (B). C, Primary root length. Plants were grown vertically on basal medium for eight days. Values indicate the means ± sd of three biological replicates, each with 8–10 plants; **P < 0.01, Student’s *t* test. D, Phenotypic comparison of 32-d-old wild-type and *sahy1* mutant plants. E, Phenotypic comparison of flower organs. Opening flowers with detached sepals and petals were obtained from plants grown in soil for 36 d. Scale bars = 100 *μ*m. F, Pollen phenotype observed by scanning electron microscopy. G, Pollen activity assay. Pollen viability was determined by FDA staining. The corresponding quantitative data (at right) were derived from the number of active pollen grains divided by total pollen grains. Arrows indicate the inactive pollen grains. Values are the means ± sd of two independent experiments, each with *n* = 400–500; **P < 0.01, Student’s *t* test. Scale bars = 200 μm. H, Comparison of siliques. Siliques attached to the flower shoots (left panel) and seed development (middle panel) are shown. Arrowheads indicate aborted/unfertilized embryos in *sahy1*. Values (right panel) are the means ± sd of 12 siliques. Two independent experiments were performed, each with *n* ≥ 12, which produced consistent results. Plants were grown in soil for ∼6 weeks. Siliques at positions 5–7 of primary shoots were used.

To better understand salt-responsive components and the pathways upon which they act, we genetically screened T-DNA insertion mutants that exhibited increased sensitivity to salt. We isolated several *salt-hypersensitive mutant*s (referred to as *sahy*s; Huang et al., 2018) by growing over 10,000 lines of T-DNA-tagged seeds (Alonso et al., 2003) that were obtained from the Arabidopsis Biological Resource Center (ABRC, OH), on half-strength MS media supplemented with both 1% sucrose (referred to as basal media hereafter) and 150-mM NaCl. The *sahy1* mutants showed unique phenotypes: they were small and exhibited salt hypersensitivity when grown on salt-supplemented media (Figure 1, A and B). Both wild-type plants and the *sahy1* mutants grew normally on basal media, although the mutants were smaller. When grown on basal media for 10 d, the first-pair leaves of the *sahy1* mutants were slightly more pointed than that of the wild-type plants (Figure 1A). On 150-mM NaCl media, 10-d-old wild-type seedlings showed steady growth with expanded cotyledons and greening leaves, whereas the *sahy1* mutant seedlings displayed salt hypersensitivity and postgermination developmental arrest (Figure 1B). The roots of *sahy1-1* and *sahy1-2* were ∼29.4% and 31% of the length of the wild-type roots (Figure 1C), respectively, when grown on basal media for 8 d. At 32 d, the *sahy1* mutant plants grown in soil were smaller than the wild-type plants and exhibited delayed flowering (Figure 1D). These data suggest that mutations in *SAHY1* alter salt sensitivity and plant growth and development.

To investigate whether the sterility of *sahy1* mutants is correlated with defects in flower structures and pollen development, we examined the flower organs and pollen grains of the plants. The wild-type flowers normally contain four petals and six stamens (four long and two short stamens). However, the majority of *sahy1* flowers contained five stamens; one short stamen was missing (Figure 1E). Occasionally, five petals and four or six stamens were observed in *sahy1* flowers (Supplemental Figure S1A). Moreover, a subset of the mutant pollen grains was indeed defective and showed deformation (Figure 1F). A pollen viability assay performed by fluorescein diacetate (FDA) staining indicated that more inactive pollen grains were produced by the *sahy1-1* mutant than the wild-type plants (Figure 1G). Consistently, pollen germination assays (Wang et al., 2008) revealed a lower germination rate in the *sahy1* mutant (50.1 ± 1.5) than in the wild-type plants (65.9 ± 1.0) and complemented transgenic plants pCOM1 (69.8 ± 1.3; Supplemental Figure S1B). In addition, the *sahy1* mutants displayed siliques that were shorter than those of wild-type and pCOM1 plants at maturity (Figure 1H, left panel). The *sahy1* plants produced fewer normal seeds and more aborted seeds than the wild-type plants (Figure 1H, right panel). The aborted seeds observed in *sahy1* plants were largely white and transparent (Figure 1H, middle panel with arrowheads), presumably due to a lack of fertilization. Taken together, these data suggest that *sahy1* sterility is associated with defects in flower organ identity and pollen grain activity.

### Complementation tests

**Figure 2:**
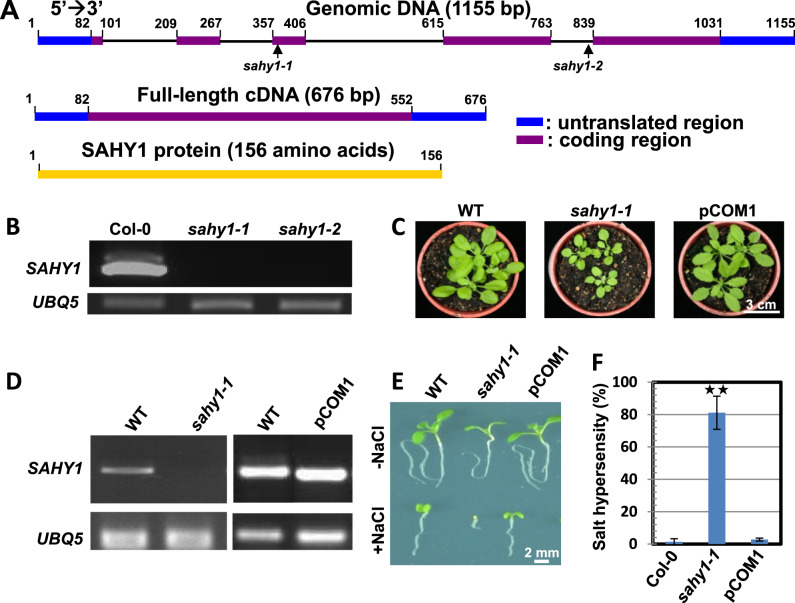
Molecular cloning and complementation test. A, Gene structure of *SAHY1*. The arrows indicate the T-DNA insertion sites in *sahy1*. B, Comparison of the *SAHY1* transcript between Col-0 and *sahy1* plants. Plants grown on basal medium for 12 d were used for RT-PCR. UBQ, ubiquitin. C, D, Complementation test. Plants were grown in soil for 23 d (C). RT-PCR showed the *SAHY1* transcript levels (D). E, Phenotypic comparison among the genotypes. Plants were grown on media with or without NaCl (150 mM) for 10 d. F, Quantitative data derived from (E). The percentage of salt hypersensitivity represents the number of seedlings with the postgermination developmental arrest phenotype versus the total number of seedlings. Values (F) are the means ± sd of three biological replicates, each with 80–100 seeds; ^**^P < 0.01, Student’s *t* test.

To better understand the molecular basis of the role of SAHY1 in plant growth and development, we cloned *SAHY1* using a genomic DNA walking approach (Sieber et al., 1995). The results indicated that the T-DNA was inserted in the third exon and fourth intron of At2g01640 in *sahy1-1* and *sahy1-2*, respectively. The full-length *SAHY1* cDNA is 676-bp long and contains an open reading frame of 471 bp that encodes a deduced protein of 156 amino acids (Figure 2A). Reverse transcription (RT)-polymerase chain reaction (PCR) analysis further indicated that the *SAHY1* transcript was present in the wild-type plants but not in the *sahy1-1* and *sahy1-2* mutant plants (Figure 2B). Therefore, both *sahy1-1* and *sahy1-2* were knockout mutants. To further confirm that the *sahy1* mutant phenotypes are caused by a defect in *SAHY1*, we performed a genetic complementation experiment. We constructed transgenes in which the *SAHY1* coding region was fused to the green fluorescent protein (GFP) coding region and driven by the *SAHY1* native promoter approximately 1.1-kbp upstream of the ATG start codon (i.e., *pSAHY1*::*SAHY1-GFP*), and then transferred this construct into the *sahy1-1* mutant plants via *Agrobacterium*-mediated transformation. The resulting homozygous transgenic plants expressing *pSAHY1*::*SAHY1-GFP* in the *sahy1-1* mutant background (named pCOM) were normal in terms of plant size (Figure 2C) and salt sensitivity (Figure 2, E and F). The *SAHY1* transcript was expressed at similar levels in pCOM1 and wild-type seedlings, whereas the *sahy1-1* mutant lacked this transcript (Figure 2D). The defects in the stamens and silique length of *sahy1* plants were rescued in the complemented transgenic plants (Figure 1, E and H). Overall, these data demonstrate that the *sahy1* mutant phenotype is due to defects in *SAHY1*.

### The SAHY1 protein is conserved across eukaryotes

The protein sequence alignment revealed that SAHY1 shows 85% identity to a protein in *Brassica napus* (XP_013720480) and 55%–61% identity to proteins in dicots and monocots, such as tobacco (*Nicotiana tabacum*, XP_016514709), tomato (*Solanum lycopersicum*, XP_004252605), rice (*Oryza sativa*, Os12g0244500), and maize (*Zea mays*, ACF86521). Furthermore, Arabidopsis SAHY1 shows 29%–38% identity to its putative orthologues in fission yeast (*Schizosaccharomyces japonicus*), unicellular green algae (*Ostreococcus tauri*), and green algae (*Chlamydomonas reinhardtii*; Supplemental Figure S2A). In phylogenetic analyses, the SAHY1-related plant proteins formed a clade separate from those of animals (Supplemental Figure S2B), including humans (*Homo sapiens*) and chimpanzees (*Pan troglodytes*). These data suggest that the SAHY1 sequence diverged when plants and animals split into separate lineages.

### SAHY1 is a nucleolar protein present predominantly in root tips, pollen grains, and developing seeds

**Figure 3:**
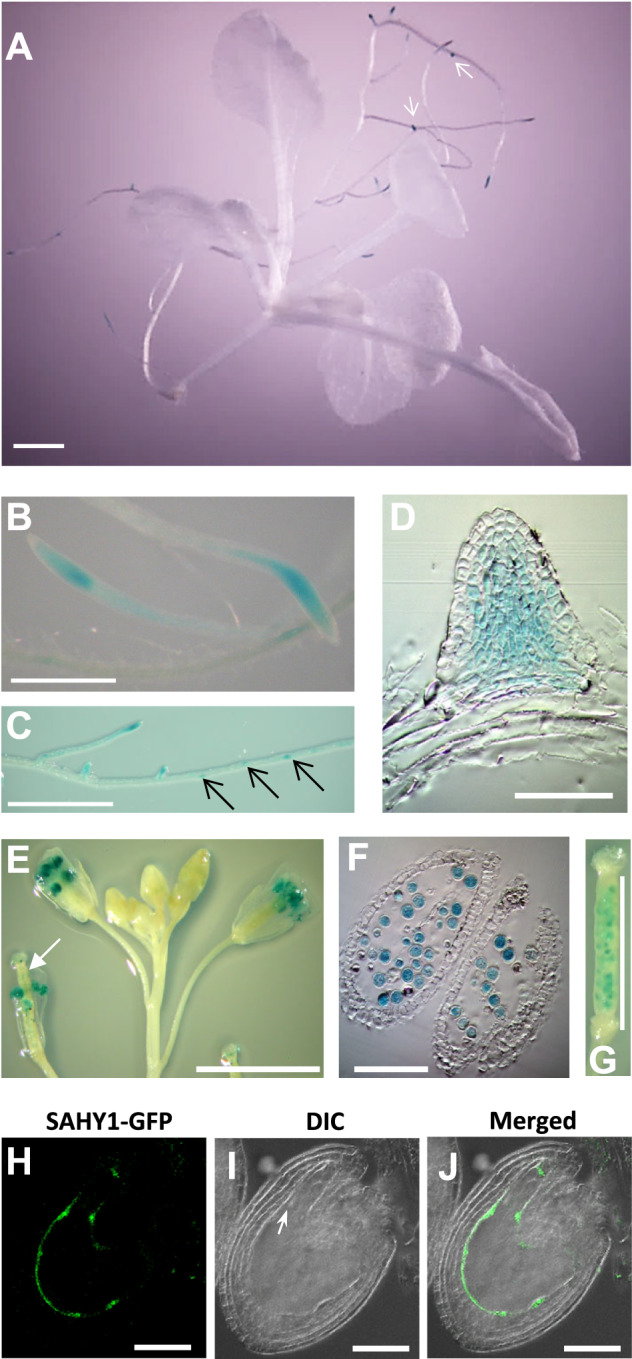
Tissue-specific expression of *SAHY1*. A–C, GUS staining of root tips and lateral roots of *pSAHY1::GUS* transgenic seedlings grown on basal medium for 12 d. Arrows indicate sites of lateral root initiation. Scale bar = 1 mm. D, Resin-embedded section of an emerging lateral root. Scale bar = 50 *μ*m. E, GUS staining in the pollen grains of opened flowers. The arrow indicates a young silique with GUS-stained developing seeds. Scale bar = 5 mm. F, Resin-embedded anther section. Scale bar = 100 *μ*m. G, GUS staining in the developing seeds within a young silique. Scale bar = 2 mm. H–J, Expression of SAHY1-GFP in developing seeds. Flowers of the complemented transgenic plants pCOM1 were hand-pollinated. The developing seeds were visualized by confocal microscopy on day 2 of pollination. Arrow indicates the early globular embryo. Scale bar = 50 *μ*m.

To determine the function of SAHY1 in specific plant tissues and subcellular compartments, we examined the spatiotemporal expression patterns of *SAHY1* and the subcellular localization of its encoded protein. Transgenic plants expressing *pSAHY1::β-glucuronidase* (*GUS*) exhibited GUS staining signal in the root tips and emerging lateral roots of seedlings (Figure 3, A–D) and in mature pollen grains (Figure 3E). Resin-embedded sections showed the GUS signal in each cell layer of emerging lateral roots, but little signal in epidermal cells (Figure 3D) and inside mature pollen grains (Figure 3F). In addition, GUS signals were also detected in the developing seeds of young siliques (Figure 3, E, arrow, and G), in which the stamens remained attached. To further examine the precise stage of the developing seeds, the complemented transgenic plants (pCOM1) were hand pollinated for 1–5 d, and the developing seeds were then visualized by confocal microscopy. The resulting GFP signal was visualized primarily in the dividing endosperm cells of developing seeds of the hand-pollinated siliques at day 2 (Figure 3, H–J), which showed an embryo at the early globular stage (Figure 3I, arrow). These data suggest that *SAHY1* is expressed predominantly in tissues with active metabolism and those undergoing cell division.

**Figure 4:**
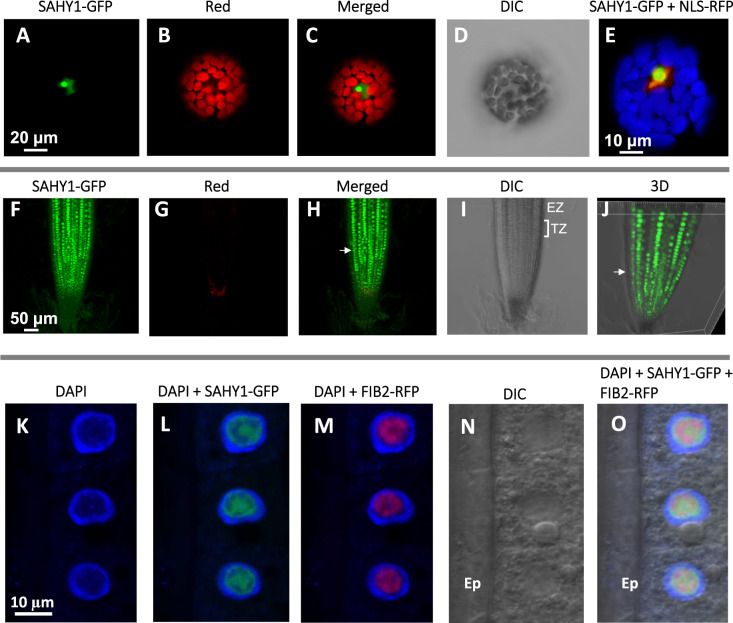
Subcellular localization of SAHY1. A–D, Arabidopsis mesophyll protoplasts expressing *35S::SAHY1-GFP*. The SAHY1-GFP signal was visualized by confocal microscopy under light with green (A) and red (B) wavelengths. The red signal in the chloroplasts is due to chlorophyll autofluorescence. Image (C) is a merged image of (A) and (B). DIC view (D) shows cell shape. E, Expression of *35S::SAHY1-GFP* and *35S::NLS-RFP* in the protoplast. Chlorophyll autofluorescence is masked and shown in blue. F–I, Localization of SAHY1-GFP in complemented transgenic plants expressing *pSAHY1::SAHY1-GFP*. The root tips of seedlings grown on basal media for 12 d were visualized. J, 3D structure. Arrows indicate the epidermal cell layer; TZ and EZ indicate the transition and elongation zones, respectively. K–O, Colocalization of FIB2-RFP with SAHY1-GFP in the root tips of the complemented transgenic plants expressing *pSAHY1::SAHY1-GFP* and *35S::FIB2-RFP*. The cortex cells were stained with 4ʹ,6ʹ-diamidino-2-phenylindole (DAPI). The seedlings were grown on agar plates for 9 d. Ep, epidermal cell layer.

Subcellular localization analyses of the *pSAHY1*::*SAHY1-GFP* transgene or both *pSAHY1::SAHY1-GFP* and a nuclear localization signal peptide fused to RFP (*35S::NLS-RFP*; Lee et al., 2001) in leaf protoplasts confirmed that SAHY1-GFP was strongly expressed in the nucleolus, whereas weak signals occurred in other regions of the nucleus (Figure 4, A–E). Transgenic plants expressing *pSAHY1*::*SAHY1-GFP* showed GFP signals primarily in the nuclei of cells in the cell division and root elongation zones (Figure 4, F–I), with the exception of cells in the epidermal cell layer (Figure 4, H and J, arrows). High magnification observations in conjunction with 4ʹ,6ʹ-diamidino-2-phenylindole (DAPI) counterstaining revealed that only the nucleoplasm was stained (Figure 4K, blue) and not the nucleolus. In contrast, SAHY-GFP signals were primarily restricted to the nucleolus and colocalized with the nucleolar marker fibrillarin 2 (i.e. FIB2-RFP; Barneche et al., 2000; Figure 4, K–O). Collectively, these data demonstrated that SAHY1 is a nucleolus-associated protein.

### Alterations in the development of embryos and the expression of auxin transport carriers in *sahy1* mutants

**Figure 5:**
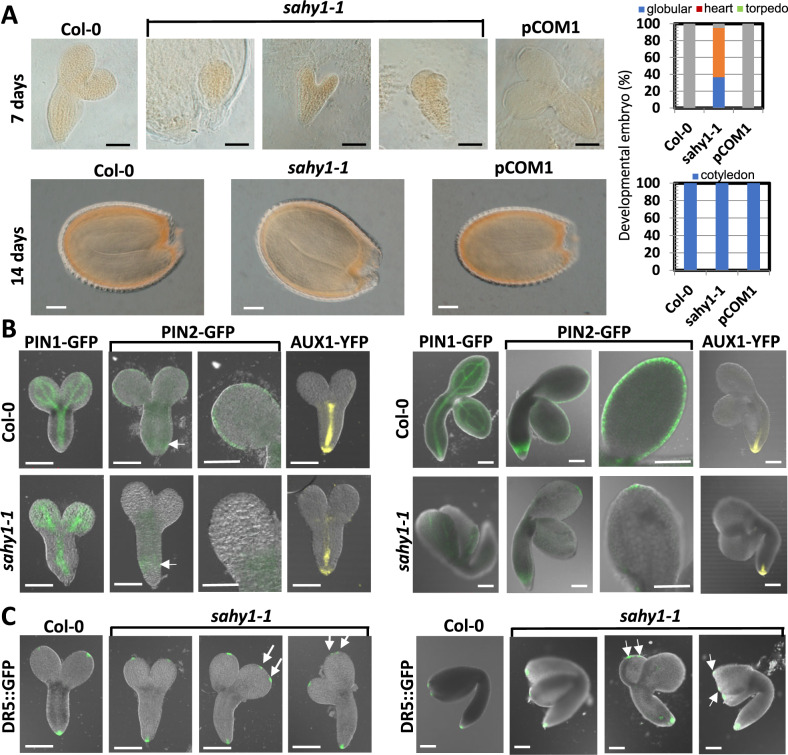
The *sahy1* mutant shows delayed embryo development and differential expression of auxin transport carriers. A, Embryo development. Plants were grown in soil for 6 weeks, followed by hand pollination of the emasculated unopened flowers. The developing siliques were then checked after 7 d (upper panel) or 14 d (lower panel). A total of 77, 41, and 132 embryos from Col-0, *shay1-1*, and pCOM1, respectively, were examined from the hand-pollinated siliques after 7 d, and more than 50 embryos of each genotype from the hand-pollinated siliques were examined after 14 d. B, C, Differential expression of auxin transport carriers and auxin-responsive marker during embryo development. Transgenic plants were grown in soil for 6 weeks, followed by the examination of the expression of auxin transport carriers (B) or the auxin-responsive marker DR5::GFP (C) in embryos at different developmental stages under a confocal microscope. The arrow indicates the GFP signal. Scale bars = 100 *µ*m (A–C).

As the transgenic plants expressing *pSAHY1::GUS* or *pSAHY1::SAHY1-GFP* also showed GUS or GFP signals during early seed development (Figure 3, G and J), we further assessed possible defects in *sahy1* embryo development. The examination of the siliques revealed that the fertilized *sahy1* seeds showed delayed embryo development. Seven days after the hand pollination of unopened emasculated flowers, we observed that the majority of the embryos of the *sahy1* mutant were at the globular and heart stages, whereas few were in the torpedo stage; instead, embryos at the torpedo stage were observed in the wild-type and pCOM1 plants (Figure 5A, upper panel). However, 14 d after hand pollination, we observed that the *sahy1-1* embryos had developed to the cotyledon stage, which was similar to that observed in wild-type and pCOM1 embryos (Figure 5A, lower panel). As embryogenesis is associated with auxin distribution and signaling, the expression of auxin transport carrier proteins was examined at both the torpedo and cotyledon stages. In general, PIN1-GFP signals were observed throughout the root stele and cotyledon vascular network at the torpedo and cotyledon stages in both the wild-type and *sahy1* plants. Notably, the PIN1-GFP signal was more discontinuous in the vascular stripes of the cotyledons of *sahy1-1* embryos than in the wild-type embryos. Interestingly, PIN2-GFP signals were observed along the cotyledon margins and in the subapical cell division zone of the roots in the wild type, whereas the signal was weakly visible both in the cell division zone of the roots and at the cotyledon terminal ends in the *sahy1* mutant (Figure 5B). AUX1-YFP fluorescence was detected in the root tips and vascular stele region in the wild-type and *sahy1* embryos; however, the signal was reduced in the latter. Although the auxin-responsive marker signal DR5::GFP was observed in the tips of both the roots and cotyledons of developing embryos in both the wild-type and mutant, multiple signal spots (6 of 12 embryos) were frequently visible on the cotyledon tips of *sahy1-1* torpedo embryos (Figure 5C, arrows); however, the frequency with which the multiple signal spots were observed decreased in the cotyledon-stage embryos (3 of 24 embryos). Based on these data, the mutation of *SAHY1* results in both delayed early embryo development and distinct expression patterns of auxin transport carriers and the auxin-responsive marker DR5::GFP. The altered expression patterns of PIN-GFP proteins and DR5::GFP are closely linked to the discontinuous development of the venation pattern in the *sahy1* embryonic cotyledons.

### Cell proliferation is impaired in *sahy1* root growth

**Figure 6:**
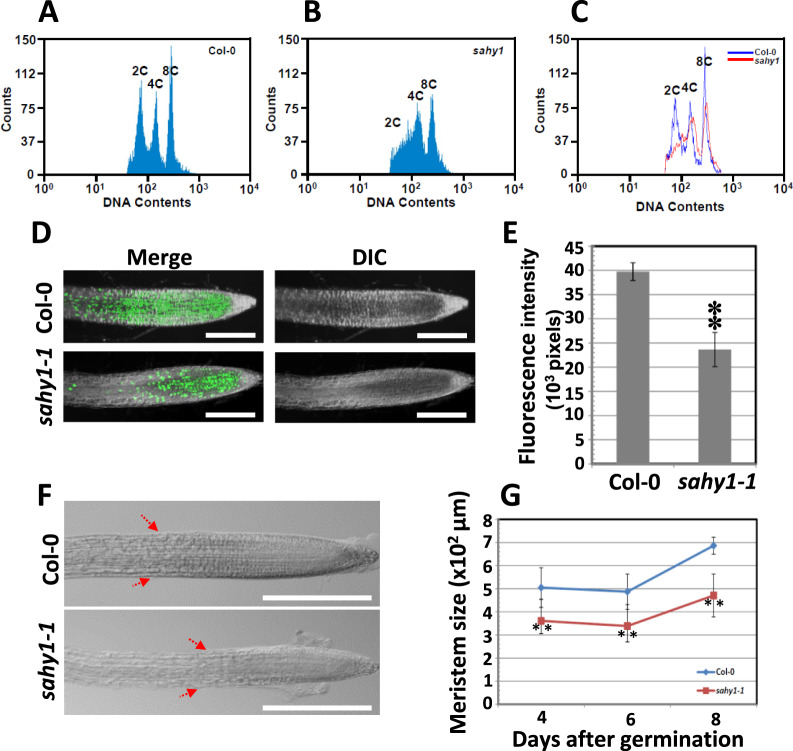
Reduction of endoreduplication and DNA synthesis in the root tip cells of the *sahy1-1* mutant. A, B, Ploidy analysis of root tip cells of wild-type (A) and *sahy1-1* (B) plants. C, Comparison of nuclear DNA content derived from (A) and (B). D and E, EdU staining. The root tips of plants grown vertically on basal medium for 8 d were subjected to EdU staining (D), and the fluorescence was quantified (E). F, Analysis of meristem size. Red arrows indicate the border region of the transition zone derived from 8-d-old root tissue. G, Quantification of meristem size between the wild-type and *sahy1*. The values in (E) and (G) are the means ± sd of two independent experiments, each with *n* = 5; ^**^P < 0.01, Student’s *t* test. Scale bars = 0.2 mm in (D) and (F).

To determine whether the short roots of *sahy1* plants are due to a defect in cell division, we measured the total genomic DNA content in the wild-type and *sahy1-1* root tips using flow cytometry. Compared with those of the wild-type, the root tips of the *sahy1-1* mutant lost the diploid (2C) peak in the root tips and showed fewer tetraploid (4C) and octoploid (8C) cells (Figure 6, A–C). Therefore, the short roots and slow growth of the *sahy1-1* mutant are related to reduce cell division in the root tips.

To further confirm this hypothesis, we stained the root tips with 5-ethynyl-2′-deoxyuridine (EdU), a marker for the S phase (the DNA synthesis phase) of cell division. The EdU fluorescence level in the *sahy1-1* root tips was ∼56.4% of that in the wild-type root tips (Figure 6, D and E), suggesting that DNA synthesis is impaired in the root tips of the *sahy1-1* mutant. In addition, EdU staining was localized primarily to the cell division zone of the root tips, and this region was longer in the wild type than in the *sahy1-1* mutant. Moreover, the root meristem size was measured as the distance from the quiescent cells to the border region of the transition zone and compared between the wild-type and *sahy1-1* plants. The meristem size of the *sahy1-1* mutant was smaller than that of the wild type after 4, 6, and 8 d of seed germination (Figure 6G). Together, these data suggest that cell proliferation is impaired within the short roots of *sahy1* mutants.

### SAHY1 is involved in pre-rRNA processing and ribosome biogenesis

**Figure 7:**
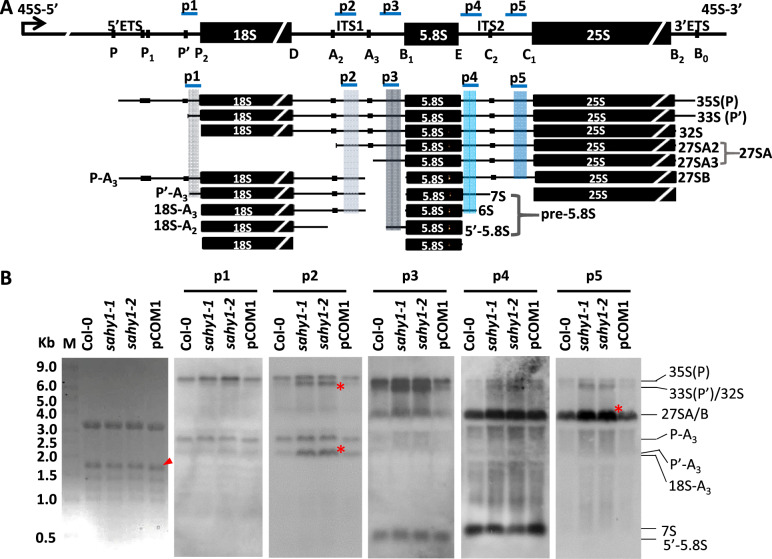
Accumulation of pre-rRNA intermediates in *sahy1*. A, Schematic diagram depicting 45S rDNA encoding 35S pre-rRNA and its processed intermediates. Longitudinal lines representing cleavage sites or pre-rRNA processing. The regions used for the probes are indicated by blue lines. The bent arrow indicates the transcription start site. This diagram is adopted from the reports by Hang et al. (2014) and Micol-Ponce et al. (2018). B, Northern blot analyses showing steady-state levels of pre-rRNA precursors. mRNA size marker; the star represents the accumulation of pre-rRNA intermediates; the arrowhead indicates 18S rRNA. Each lane contains 10 *µ*g RNA. The plants were grown vertically on basal media for 12 d and then harvested for Northern blot analysis.

The nucleolus has two primary functions, namely rRNA production and ribosome biogenesis/assembly. The 45S/35S pre-rRNA contains various sequences, including a 5ʹ-ETS, ITS1, and ITS2, and a 3ʹ-ETS, that undergo rRNA processing (Figure 7A). To examine whether the nucleolar protein SAHY1 plays a role in pre-rRNA processing, Northern blot analyses were performed to determine the abundance of pre-rRNA intermediates by hybridization with specific probes (Supplemental Table S2) located within the ETS or ITS regions (Zhu et al., 2016). As shown in Figure 7B, intermediates of 33S(P′)/32S, 27SA/B, and 18S-A3 accumulated at higher levels in the *sahy1* mutants than in the wild-type plants (Figure 7B, asterisks), whereas the levels of the total mature 18S rRNA in *sahy1* mutants were similar to those in the wild type (Figure 7B, arrowhead). The accumulation of pre-RNA intermediates in the complemented transgenic pCOM1 plants was largely restored to the normal level found in wild-type plants. These results suggest that SAHY1 plays an important role in pre-rRNA processing.

**Figure 8:**
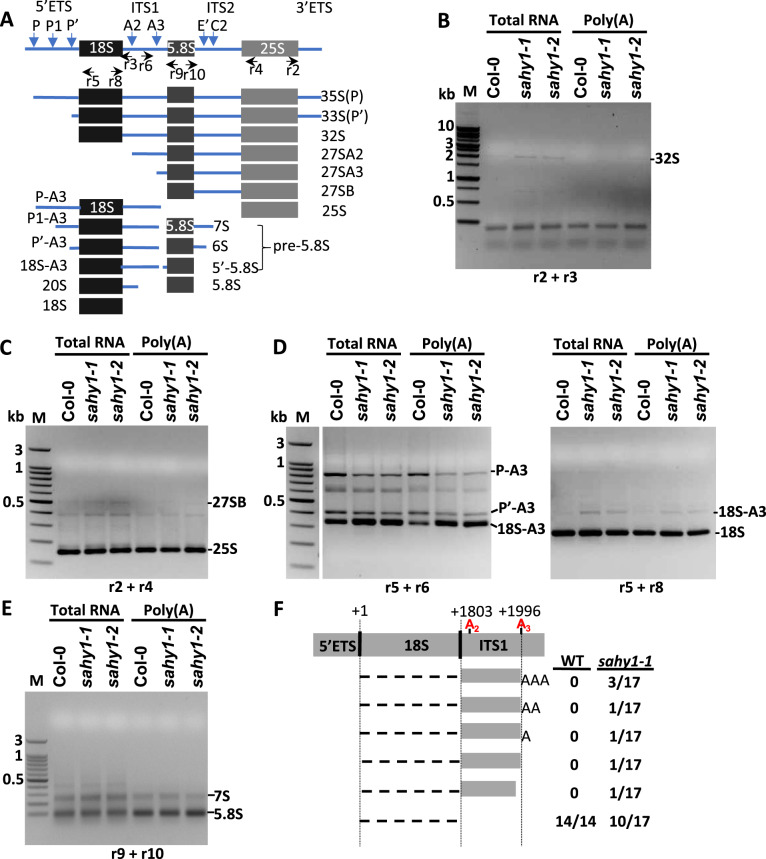
Circular RT-PCR and sequencing of 18S pre-rRNA intermediates. A, Schematic diagram showing circular 35S pre-rRNA intermediates and primer positions for cRT-PCR amplification. This diagram is adopted from Micol-Ponce et al. (2018). B–E, DNA gels showing cRT-PCR products. Each lane contains 10 *µ*l of cRT-PCR products. M, marker. F, A schematic diagram indicating the results of 18S-A3 pre-rRNA flanking sequences of cRT-PCR cloning. The cRT-PCR product for cloning was derived from (D) using r5, r8 primer pair.

Furthermore, circular RT-PCR (cRT-PCR) was used to determine the sequences of the unprocessed pre-rRNAs at both ends based on specific primer pairs (Figure 8A). The r2, r3 primer pair may amplify 35S(P), 33S(P′), and 32S pre-rRNAs through cRT-PCR. However, the cRT-PCR products only displayed an accumulated band representing 32S in the total RNA samples of *sahy1* mutants (Figure 8B). Similarly, the 27SB pre-rRNA was slightly accumulated in the total RNA of *sahy1* plants using the r2, r4 primer pair (Figure 8C). When the r5, r6 primer pair was used, the cRT-PCR products revealed the accumulation of the 18S-A3 pre-rRNA in *sahy1*, but the P-A3 and P′-A3 pre-rRNAs were reduced in both total RNA and poly(A) samples (Figure 8D, left panel). Consistently, the accumulation of the 18S-A3 pre-rRNA was observed in *sahy1* when the r5, r8 primer pair was used (Figure 8D, right panel). However, the 7S pre-rRNA showed no difference between the wild-type and *sahy1* plants (Figure 8E) when the r9, r10 primer pair was used. In this study, the banding patterns of the cRT-PCR products of the 25S, 18S, and 5.8S rRNAs were similar between the wild type and *sahy1-1* (Figure 8, C–E). Furthermore, the cloning and sequencing of 18S cRT-PCR products using the r5 and r8 as primers showed that the sequences from the wild-type contained mature 18S sequences at both ends. However, in addition to the mature 18S sequence, the *sahy1* mutants contained unprocessed sequences in the ITS1 region between sites A_2_ and A_3_ (Figure 8F), which appeared with or without (poly)adenylation at the terminal end. These data further confirm the involvement of SAHY1 in the removal of ITS1 sequences (the ITS1-first pathway) during 18S pre-rRNA processing.

In the Arabidopsis genome, there are several hundred copies of the 45S rDNA gene, which contains four variants (*VAR*s) in their 3′-ETS sequences, and their expression can be regulated spatiotemporally by RBFs, such as nucleolin and RRP7 (Pontvianne et al., 2007; Micol-Ponce et al., 2018). To examine whether SAHY1 affects the expression of the 45S rDNA gene, RT-PCR was employed to determine the 45S rDNA 3′-ETS and the corresponding transcripts. The 45S rDNA gene showed a similar pattern among the wild type, *sahy1* mutants, and pCOM1 plants, in which *VAR1* was the most abundant (Supplemental Figure S3A, left panel). Although the 45S rRNA also showed a similar pattern among the genotypes tested, *sahy1-1* exhibited a slightly higher level of *VAR1*. However, the *VAR1* level in *sahy1-2* was close to those of the wild-type and pCOM1 plants (Supplemental Figure S3A, right panel). The cause of this discrepancy between *sahy1-1* and *sahy1-2* is unknown. However, as Northern blot and cRT-PCR analyses revealed similar defects in pre-rRNA processing in these two allelic mutants (Figures 7 and 8), we thus suggest that the effect of SAHY1 on the expression of the 45S rDNA gene is minor.

Defects in pre-rRNA processing might affect ribosome biogenesis, with potential effects on ribosome abundance. Thus, the ribosomal subunit population was analyzed using sucrose gradients. Compared with those in the wild-type and pCOM1 plants, the levels of the 40S and 60S ribosome subunits in the *sahy1* mutant plants showed no significant difference. However, the level of the 80S subunit was ∼21%–24% higher in *sahy1* than in the wild type and pCOM1 (Supplemental Figure S3, B and C). These data suggest that SAHY1 plays a role in ribosomal subunit assembly to form 80S ribosomes.

### SAHY1-associated proteins revealed by Co-IP

To better understand the function of SAHY1 in ribosome biogenesis, Co-IP coupled with tandem mass spectrometry (Co-IP MS/MS) was used to identify SAHY1-associated proteins or complexes. SAHY1-GFP derived from the complemented transgenic plant expressing *pSAHY1::SAHY1-GFP* (i.e. pCOM1) was not detected by GFP antibodies in the immunoblots, presumably due to the low abundance of the SAHY1 protein in *Arabidopsis*. To enhance the expression of the SAHY1 protein, we generated transgenic plants constitutively expressing *SAHY1-Myc* driven by the *35S* promoter. The SAHY1-Myc fused protein could be recognized by a monoclonal Myc antibody in immunoblot analysis (Supplemental Figure S4). Although SAHY1 was expressed in the nucleolus and nucleus (Figure 4), SAHY1-Myc was detectable in the total proteins in the immunoblot analysis (Supplemental Figure S4C). This result indicates that some of the nuclei might have been disrupted during total protein extraction. Thus, in this experiment, both total and nuclear proteins were separately extracted from wild-type (Col-0) and SAHY1-Myc-overexpressing transgenic plants, followed by a Co-IP MS/MS assay. The selected SAHY1-Myc-associated proteins were exclusively present in the transgenic plants and not in the wild-type plants. In addition, these candidate proteins were present in at least the nucleolus or nucleus based on the Gene Ontology (GO) cellular components defined by The Arabidopsis Information Resource (TAIR) Araport11 annotation except for RPS4A (At2g17360), which was localized in the cytosol or cytosolic SSU processome. Accordingly, at least 25 SAHY1-Myc-associated proteins were identified (Supplemental Table S1), including seven large RPs (RPL10, two RPL6s, RPL2, RPL11, L28e, and RPL6A) and four small RPs (RPS18A, RPS19e, and two RPS4As), six RBPs (such as LOS1, nucleolin like 1, transducin/WD40 repeat-like protein, homolog of NAP57, P-loop hydrolase and RAN2), and other proteins (such as CRU1, HSP90.7, J3, RBGB3, and GBPL3) whose functions in ribosome biogenesis remains to be determined. Collectively, these data support the involvement of SAHY1 in the biogenesis of small and large ribosomal subunits through its interacting proteins/complexes involved in endonucleolytic cleavage and nucleotide modification during pre-rRNA processing. It is also likely that SAHY1 functions in preribosomal particle export from the nucleus via RAN2 activity.

### Gene expression profiles mediated by *SAHY1*

We further determined the gene expression profiles of the root tissues of wild-type and *sahy1-1* plants grown on basal media for 12 d using Affymetrix ATH1 GeneChips. The expression of 286 genes was altered in the *sahy1-1* mutant (169 up- and 117 downregulated), with a signal fold change >2. The genes whose expression was altered are involved in various functions, including metabolic processes, stress responses, protein modification, cell wall organization, transcriptional regulation, and signal transduction. Interestingly, at least 10 of the differentially expressed genes were involved in ribosome biogenesis/assembly (Table 1), all of which were upregulated in the mutant. Six of the 10 genes were verified by RT-quantitative PCR (RT-qPCR; Supplemental Figure S5). These data further suggest that the dysfunction of SAHY1 alters the expression of *RP* and *RBF* genes under normal growth conditions.

**Table 1:** Differentially expressed genes related to ribosome biogenesis/assembly in *sahy1*

AGI number	Description	Fold change^a^	GO molecular/biological function
**AT3G49910** ^b^	Translation protein SH3-like family protein	2.01	Structural constituent of ribosome
**AT5G59240**	Ribosomal protein S8e family protein	2.13	Structural constituent of ribosome
AT5G44710	Ribosomal protein S27/S33, mitochondrial	2.29	
**AT5G48760**	Ribosomal protein L13 family protein	2.72	Structural constituent of ribosome
**AT1G17560**	Ribosomal protein HLL, mitochondrial	3.05	Structural constituent of ribosome
AT1G17960	Threonyl-tRNA synthetase	3.77	Translation
**AT2G40010**	Ribosomal protein L10 family protein	3.89	Structural constituent of ribosome
AT2G18720	Translation elongation factor EF1A	8.26	Translation factor activity
**AT3G28500**	60S acidic ribosomal protein family	9.06	Structural constituent of ribosome
AT3G18610	ATNUC-L2 (NUCLEOLIN-LIKE 2)	18.17	Nucleotide binding

a Fold changes in the mutants are presented as the means of three biological experiments and are normalized to those of the corresponding wild-type.

b Bold type represents the genes verified by RT-qPCR (Supplemental Figure S5). The raw data are available in the GEO database under accession No. GSE49919.

### *sahy1* Mutants exhibit increased tolerance to protein translation inhibitors

As aforementioned, SAHY1 is involved in ribosome biogenesis that is essential for protein translation. To examine whether SAHY1 also affects protein translation, *sahy1-1* mutant plants were treated with streptomycin, an aminoglycoside antibiotic that inhibits protein translation in prokaryotes, and cycloheximide (CHX), a translational inhibitor. The *sahy1-1* mutant plants were more resistant to streptomycin (50 *μ*g/mL) than the wild-type plants (Supplemental Figure S6, A and B). Under these growth conditions, bleached cotyledons were observed in 68.0 ± 14.5% and 21.1 ± 1.6% of the wild-type and *sahy1-1* plants, respectively (Supplemental Figure S6C). When grown on basal media, the wild-type plants were larger than the *sahy1*-*1* mutants (Supplemental Figure S6D). However, the wild-type plants were similar in size to the *sahy1-1* plants and showed more chlorotic leaves (65.9%) than did the mutants (11.7%; Supplemental Figure S6, E and F) when they were grown on 3.6-*µ*M CHX-supplemented agar plates. These data suggest that the *sahy1-1* mutant is more tolerant than the wild-type to streptomycin and CHX.

## Discussion

### SAHY1 is involved in pollen and embryo development and cell proliferation

Ribosomal mutants defective in 18S rRNA processing frequently show effects on gametogenesis and embryo development, processes that are characterized by a high demand for ribosome biogenesis for the translation of proteins essential for cell division and growth (Missbach et al., 2013). Because of the importance of maternal effects, the disturbance of female gametogenesis is often lethal (Yadegari and Drews, 2004) and is frequently accompanied by disrupted gamete transmission observed in F2 segregating populations, as observed in the *rps5a*, *rrp5*, *nof1*, *rh36*, and *swa1* mutants (Weijers et al., 2001; Shi et al., 2005; Harscoët et al., 2010; Huang et al., 2010; Missbach et al., 2013); these mutants only survive as heterozygotes for phenotypic observation. However, some mutants defective in 18S pre-rRNA processing, such as *sahy1* (in this study) and *apum23* (Abbasi et al., 2010), are viable as homozygotes but are partially sterile and display short siliques and reduced seed numbers. This type of sterility is likely associated with disturbed pollen and/or embryo development. In the present study, in addition to decreased stamen numbers and smaller anthers, the mutation of *SAHY*1 resulted in a relatively greater proportion of pollen grains with aberrant shapes and reduced viability and germination, suggesting that the sterility of *sahy1* is associated with the abnormal development of pollen grains and structural changes in flower organs.

Early stage embryo development is characterized by active asymmetric cell division and cell growth, which requires a large amount of functional ribosomes for protein translation and cell proliferation. *pSAHY1::SAHY1-GFP* was expressed in the dividing endosperm cells during early seed development (Figure 3, H–J), and the mutation of *SAHY1* delayed early embryo development (Figure 5A), implying that the endosperm cells may provide essential nutrients and signals for early embryo development, as reported previously (Hehenberger et al., 2012). Delayed embryo development is also observed in other ribosome-related mutants, such as heterozygous *rps5a*, *noc4*, *nob1*, and *yao* mutants (Weijers et al., 2001; Missbach et al., 2013). This phenomenon suggests that SAHY1 and other proteins might play important roles in the production of sufficiently functional ribosomes for cell division and early embryo development. The phytohormone auxin plays important roles in controlling early embryo growth and development (Jenik et al., 2007; Möller and Weijers, 2009). The direction of auxin polar transport predicted by the localization of auxin transport carriers may correspond to the expression pattern of auxin-responsive genes. In this study, the most frequently altered expression pattern observed among the tested auxin transport carriers was the expression of PIN2-GFP, which was expressed along the cotyledon margins in the wild-type embryos at both the torpedo and cotyledon stages (Figure 5B), whereas PIN2-GFP signals were restricted to the tips of the cotyledons in the *sahy1* embryos. The expression of the auxin responsive marker DR5::GFP in *sahy1* cotyledons consistently differed from wild-type cotyledons (Figure 5C), reflecting differences in auxin transport, and signaling during embryo development and the vascular formation of embryonic cotyledons between wild-type and *sahy1* cotyledons. Most ribosome-related mutants and auxin transport inhibitor-treated plants also exhibit defects in leaf and/or cotyledon vascular patterning. These abnormal phenotypes are attributable to defects in auxin distribution and sensing (Figure 5; Gälweiler et al., 1998; Sieburth, 1999; Petrica and Nelson, 2007; Degenhardt and Bonham-Smith, 2008; Abbasi et al., 2010; Rosado et al., 2010). Notably, an increased discontinuity of vascular patterning was observed in *sahy1* embryos at the torpedo and cotyledon stages, and was associated with the altered expression of auxin transport carriers and the auxin-responsive marker DR5::GFP (Figure 5). Thus, a defect in vascular patterning is closely linked to changes in auxin transport and signaling in the ribosome-related mutants.

The involvement of ribosome biogenesis in the cell cycle has been characterized in both yeast and animals (Tsai and Mckay, 2002; Boisvert et al., 2007; Hernandez-Verdun, 2011; Blank et al., 2017), but less evidence is available for the vegetative tissues of higher plants, particularly in root tissue. To date, researchers have reported that Arabidopsis TORMOZ (TOZ), RPS5, ROOT INITIATION DEFECTIVE 2 (RID2), OLIGOCELLUA2 (OLI2), and G-PATCH DOMAIN PROTEIN 1 (GDP1) participate in cell division. Notably, *toz* and *rps5* mutants produce embryos that exhibit aberrant cell division and arrested embryo development (Weijers et al., 2001; Griffith et al., 2007). The *rid2* mutants exhibit reduced callus induction (Ohbayashi et al., 2011), and *oli2* and *gdp1* mutants exhibit reduced leaf cell numbers (Kojima et al., 2018). In the present study, the short roots of *sahy1* plants were associated with reduced mitotic activity and DNA synthesis during S phase (Figure 6), leading to a small meristem size in the root tips. This finding suggests that SAHY1 plays a role in cell proliferation in root tissues. In addition to its effect on vascular patterning, the plant hormone auxin also plays an important role in mediating root growth and patterning (Blilou et al., 2005), and auxin transport and sensing are accordingly altered in the ribosome-related mutants. Thus, the possible regulatory mechanism is proposed that the mutation of these RP or RBP genes, including *SAHY1*, alters the ribosomal composition and biogenesis, which further results in the preferential or selectable translation of specific auxin-mediated transporters and/or responsive genes (in this study; Abbasi et al., 2010; Rosado et al., 2010; Zhao et al., 2015). The reduced translation of auxin-specific genes is regulated by the upstream protein in the auxin signaling pathway, such as *AtbZIP11* and *ARF* transcripts. Translation of these transcriptional factor proteins is downregulated via upstream open reading frame in the leader sequences of the associated mRNAs, which further attenuates the expression of their downstream targets (Kim et al., 2004; Nishimura et al., 2005; Zhou et al., 2010; Rosado et al., 2012). In this scenario, the expression of *PIN* or *AUX* transcriptions should be downregulated. However, our microarray analysis did not show reduced transcription of *PIN1* and *PIN2* or *AUX1* in *sahy1* mutants, which has also been reported in the *rpl4d* mutant, except for *PIN2* transcript that shows downregulation in the latter mutant (Rosado et al., 2012). Thus, additional studies are essential to confirm the mechanisms underlying the differential translation of auxin transport carrier proteins and auxin-responsive proteins between ribosome-related mutants and wild-type plants.

To date, most studies of ribosome biogenesis/assembly have focused on the functions of RPs or RBFs in association with plant growth and development. Little is known about the mechanisms underlying ribosome biogenesis in response to abiotic stress. As the expression of RPs or RBFs may be altered under abiotic stress (Sormani et al., 2011; Liu et al., 2012; Wang et al., 2013; Huang et al., 2018), researchers have proposed that the nucleolus or ribosome may act as a central hub to sense stress or metabolites by adjusting ribosome biogenesis and protein translation for eukaryotic cell fitness (Pechmann et al., 2013; Kalinina et al., 2018; van der Horst et al., 2020). In this regard, dysfunction of RPs or RBFs due to genetic interruption or deleterious environments will alter the ribosome composition and function, leading to changes in plant sensitivity to stress. Thus, we were not surprised that our previous genetic screening of T-DNA-tagged seeds under salt-stress conditions identified 10 *sahy* mutants. Of these mutants, at least *sahy1* (in this study) and *sahy9*, an *apum23* allele (Huang et al., 2018), reveal salt hypersensitivity and defects in ribosome biogenesis. Therefore, further studies of these *sahy* mutants will provide better insights into both ribosome biogenesis and its response to abiotic stress in the future.

### SAHY1 is a nucleolar protein involved in pre-rRNA processing and ribosome biogenesis

The dynamic nucleolus is the largest subcompartmental structure in the nucleus. The nucleolus is involved primarily in the production of rRNA and the biogenesis/assembly of ribosomes, which are responsible for protein translation in the cytosol (Venema and Tollervey, 1999; Fromont-Racine et al. 2003). SAHY1 is not present in the Arabidopsis Nucleolar Protein Database (http://bioinf.scri.sari.ac.uk/cgi-bin/atnopdb/home; Brown et al., 2005; Pendle et al., 2005) or other datasets of the nucleolar proteome (Palm et al., 2016; Montacié et al., 2017). In addition, the TAIR annotation based on bioinformatics data (Cheng et al., 2017) indicates that SAHY1 (At2g01640) is an unknown protein involved in ribosome biogenesis; however, its biological process and molecular function annotations are unknown. Because SAHY1-GFP localized to the nucleolus and weakly localized to the nucleus (Figure 4), SAHY1 appears to be a previously uncharacterized nucleolar protein in *Arabidopsis*. To date, several RBFs involved in 18S rRNA processing for 40S ribosome biogenesis, such as APUM23, NOF1, RH36, SLOW WALKER1 (SWA1), Rrp5, and Rrp7, have been reported in *Arabidopsis* (Shi et al., 2005; Abbasi et al., 2010; Harscoët et al., 2010; Huang et al., 2010; Missbach et al., 2013; Micol-Ponce et al., 2018). Because these proteins function at different steps of the mature 18S rRNA processing pathway, their dysfunction leads to the accumulation of different unprocessed 18S pre-rRNA intermediates. In addition, 33S(P′)/32S pre-rRNA frequently accumulates in most of these mutants (in this study; Abbasi et al., 2010; Missbach et al., 2013), presumably due to a general delay in pre-rRNA processing. In this study, the mutation of *SAHY1* resulted in the accumulation of unprocessed 32S, 27SB, and 18S-A3 pre-rRNA intermediates (Figures 7 and 8). Furthermore, in the complemented transgenic plants, these defects were largely rescued, suggesting that SAHY1 plays a critical role in facilitating the cleavage of the ITS1 spacer at sites A2 and A3. However, despite the defects in 18S pre-rRNA processing, the abundance of mature 18S rRNA in *sahy1* did not significantly change compared with that in the wild-type plants, reflecting the possible existence of an alternative pathway to ensure the sufficient production of mature 18S rRNAs for the biogenesis of the 40S ribosome subunit. Interestingly, the mutation of Arabidopsis *protein arginine methyltransferase 3* (*PRMT3*) also accumulates the unprocessed 32S, 27SB, and 18SA3 pre-rRNA intermediates (Hang et al., 2014), suggesting that PRMT3 and SAHY1 may have common function in pre-rRNA processing. The processing steps involved in rRNA biogenesis mainly occur in the nucleolus, but the expression of PRMT3 is localized to the cytosol. One possibility is that the methylated targets of PRMT3 may translocate into the nucleolus and interact with the SAHY1-associated subcomplex for pre-rRNA processing. In addition, the ribosomal subunit profiling reveals the reduced abundance of 60S and 80S but the increased abundance of polysomes in *prmt3* mutants compared to the wild-type plants, whereas the abundance of 80S was increased in the *sahy1* mutant (in this study). Thus, these data imply that PRMT3 and SAHY1 have similar functions in pre-rRNA processing, but they at least have different roles in ribosome assembly or activity.

Because ribosome biogenesis includes pre-rRNA processing, RP incorporation, and the participation of RBFs, defects at any step of pre-rRNA processing might alter RP and RBF expression and the ribosome composition, which may further affect ribosome function. For instance, APUM23, AtNuc-L1, and RPL4a participate in pre-rRNA processing and ribosome biogenesis (KojiMa et al., 2007; Petrica and Nelson, 2007; Pontvianne et al., 2007; Abbasi et al., 2010; Rosado et al., 2010). The mutation of these genes results in insensitivity to the protein translational inhibitors streptomycin (Supplemental Figure S6; Abbasi et al., 2010; Horiguchi et al., 2011). CHX is a eukaryotic protein biosynthesis inhibitor; although its precise functional mechanism is yet to be fully understood, it has been shown to inhibit translation elongation by binding to the E-site of the 60S ribosomal unit and interrupting the deacetylated tRNA (Pestova and Hellen, 2003; Schneider-Poetsch et al., 2010). Upon CHX treatment, Arabidopsis *apum23* and *nug2* mutants are more sensitive to CHX (Abbasi et al., 2010; Im et al., 2011), but the *rpl4a* mutant shows similar sensitivity to wild-type plants (Rosado et al., 2010). However, the *sahy1* seedlings were insensitive to CHX (Supplemental Figure S6). This discrepancy in the sensitivity or tolerance of ribosome-related mutants to antibiotics is presumably due to changes in ribosomal composition/activity and/or an aberrant ribosomal population, which may lead to the high- or low-efficiency binding of these antibiotics to ribosomes (Abbasi et al., 2010; Rosado et al., 2010). In addition, the *sahy1* mutant exhibited an unbalanced ribosomal subunit population (Supplemental Figure S3, B and C) and upregulated expression of ribosome structural components and RBF genes (Table 1), which further supports the existence of changes in ribosome composition/activity and populations.

Our current knowledge regarding pre-rRNA processing and ribosome biogenesis has been largely obtained from studies in yeast (*Saccharomyces cerevisiae*; Fatica and Tollervey, 2002; Woolford and Baserga, 2013). The assembly/biogenesis of the yeast 40S ribosomal subunit is performed by a large ribonucleoprotein complex termed the SSU processome or 90S preribosomal particle. This SSU processome is cotranscriptionally assembled during the transcription of 45S rDNA by the stepwise recruitment of RBFs. These RBFs frequently assemble as subcomplexes before incorporation into the SSU processome; for instance, small nucleolar ribonucleoprotein complexes (snoRNPs) have specific functions in nucleolytic cleavage and base modification of pre-rRNA (Sáez-Vasquez and Delseny, 2019). To search for possible yeast homologs, SAHY1 protein sequences were analyzed with the National Center for Biotechnology Information (NCBI) BLAST tool (https://blast.ncbi.nlm.nih.gov/Blast.cgi). The results indicated that SAHY1 is a Slx9p-like protein that exhibits ∼26% similarity and 13% identity to yeast Slx9p, also known as Slx9 (Fischer et al., 2015). Slx9p acts as an RBF and is involved in 18S pre-rRNA processing. The deletion of *Slx9p* in yeast causes the accumulation of 21S and 27S-A2 pre-rRNAs, which are associated with mild growth reduction. However, Slx9p is yeast specific and is not present in higher eukaryotes (Bax et al., 2006). Furthermore, it was proposed that Slx9p may bind to RanGTP and Rio2 to facilitate the loading of Crm1, a nuclear export receptor, to form a transport complex that further exports cargos from the nucleus to the cytosol (Fischer et al., 2015). Our Co-IP studies revealed that the SAHY1-associated protein RAN2 shows GTPase activity, and has been proposed to have nucleocytoplasmic transport activity (Ma et al., 2007). In addition, several RBFs were identified as SAHY1-associated proteins. For instance, LOS1, which encodes a translation elongation factor involved in cold-induced translation, catalyzes the GTP-dependent ribosomal translocation step during translation elongation (Guo et al., 2002); NUC-L1 is the primary form of Arabidopsis nucleolins involved in pre-rRNA processing and ribosome biogenesis (KojiMa et al., 2007; Pontvianne et al., 2007); NAP57 is a component of the box H/ACA snoRNP complex, which might function in the site-specific cleavage and base pseudouridylation of pre-rRNA (Filipowicz and Pogacic, 2002). Based on the GO biological functions (Gaudet et al., 2011), P-loop hydrolase might function in the assembly of box C/D snoRNP involved in the 2′-O-ribose methylation of rRNA. Based on the information provided above, we propose that the SAHY1 protein interacts with nucleolin–U3 snoRNP complex (Petrica and Nelson, 2007; Sáez-Vasquez and Delseny, 2019) and perhaps other complexes for the processing of 35S pre-rRNA at 5′-ETS and ITS1. Interruption of SAHY1 function promotes the processing of the minor pathway (5′-ETS-first pathway) and inhibits the A2/or A3 cleavage activity (ITS1-first pathway), leading to the unbalanced pre-rRNA intermediates. The interaction of SAHY1 with NAP57-box H/ACA and P-loop hydrolase-box C/D snoRNP complexes facilitates the base pseudouridylation and methylation of pre-rRNA, respectively, for the biogenesis of mature rRNA. SAHY1–LOS1 interaction facilitates the proper formation of pre-ribosome particles, which, together with base modification of pre-rRNA, are essential for translation efficiency and capacity of mature ribosomes (Sáez-Vasquez and Delseny, 2019). Thus, the mutation of SAHY1 affects ribosome composition/or activity, which is further essential for protein translation. Notably, SAHY1 was associated with several 60S RPs (Supplemental Table S1), suggesting that, in addition to facilitating the biogenesis of the 40S subunit, SAHY1 is also involved in the incorporation of these RPs into the 60S preribosomal particle or 40S and 60S preribosomal particle export from the nucleus. Additional studies are needed to confirm whether SAHY1 has a conserved function similar to that of yeast Slx9p in preribosomal particle export from the nucleus.

In conclusion, our findings demonstrate that SAHY1 is not an essential RBF, but it plays an important role in normal plant growth and development in association with auxin transport and signaling. In addition to its functions in pre-rRNA processing and ribosome biogenesis, SAHY1 might play a role in preribosomal particle export from the nucleus, similar to yeast Slx9p. These data may provide evidence of the conservation and diversification of SAHY1 function between plants and yeast.

## Materials and methods

### Plant materials and growth conditions

Arabidopsis (*Arabidopsis thaliana*) ecotype Columbia (Col-0) was used in this study. All seeds were sterilized and subjected to cold pretreatment for three days at 4°C in the dark. Seeds were sown on agar plates composed of half-strength MS basal salts, B5 organic compounds (Gamborg, 1968) and 0.05% (w/v) 2-(N-morpholino)ethanesulfonic acid monohydrate (MES). The media were supplemented with 1% (w/v) sucrose (referred to as basal media) or 150 mM NaCl. The seeds were germinated on agar plates or in soil at 22°C with a 16 h/8 h light/dark photoperiod and a light intensity of ∼80 *μ*mol m^−2^s^−1^.

### Isolation of mutants and transgenic plants

Approximately 10,000 lines of T-DNA-tagged seed pools (Alonso et al., 2003) were requested from the ABRC (OH) and over 400,000 seeds were grown on basal media supplemented with 150 mM NaCl. The seeds germinated, but further root and shoot development was inhibited; thus, these seedlings were referred to as *sahy*s. Several *sahy* mutants were obtained from this genetic screen (Huang et al., 2018). Of these mutants, *sahy1* grew slowly both on agar plates and in the soil and was thus further characterized. The *sahy1-2* mutant (GK_152B07) was obtained from GABI-Kat (http://www.GABI-Kat.de; Kleinboelting et al., 2012). The *sahy1* mutant alleles were identified by thermal asymmetric interlaced-PCR amplification of T-DNA flanking sequences (Siebert et al., 1995), followed by the sequencing of the PCR products. For tissue-specific expression, the *SAHY1* promoter region 1,110 bp upstream of the ATG start codon was amplified by PCR and fused to a *GUS* coding region to generate *pSAHY1*::*GUS* in a pSMAB704 binary vector. For subcellular localization analyses, transgenes comprising the *SAHY1* coding region fused to *GFP* were driven by the native *SAHY1* promoter (*pSAHY1*::*SAHY1-GFP*) in a pSMAB704 vector or by the *35S* promoter (*p35S*::*SAHY1-GFP*) in a pHBT95 vector. For Co-IP, the *SAHY1* coding region fused to 10× *Myc* was driven by the *35S* promoter (*35S::SAHY1-Myc*) in the Gateway binary vector pGWB520 (Nakagawa et al., 2007). The aforementioned constructs were all confirmed by sequencing and subsequently transformed into wild-type plants for overexpression or *sahy1-1* mutant plants for complementation analysis via *Agrobacterium*-mediated transformation (Clough and Bent, 1998). The homozygous transgenic lines of each construct were screened based on their resistance to an herbicide (glufosinate ammonium) or hygromycin.

### GUS staining

Transgenic plants expressing *pSAHY1::GUS* were grown on basal media for 12 d or in soil for 5 weeks. The plants or tissues were harvested and subjected to GUS staining as described previously (Hwang et al., 2012).

### Examination of embryo development

For the analysis of embryo development, emasculated unopened flowers were hand-pollinated for 7 or 14 d. Then, the developing seeds were removed from hand-pollinated siliques. For the examination of auxin carriers fused with florescent tags, the developing seeds were visualized under a confocal microscope. For the examination of embryo development, the developing seeds were cleared with a chloral hydrate:glycerol:water solution (8:1:2) for several days. Subsequently, embryos within seeds were visualized using a light microscope.

### Microarray analysis

Arabidopsis seeds were grown on basal media for 5 d, after which they were transferred to fresh media and grown vertically for another seven days. After 12 d of culture, the roots were removed and subjected to RNA extraction. cDNA synthesis and labeling, hybridization to ATH1 GeneChips, and data scanning were performed as described previously (Cheng et al., 2009). The statistical analyses included a cutoff value of *P *< 0.05, unpaired *t* test, asymptotic *P*-value computation, and multiple testing correction according to the Benjamini–Hochberg false discovery rate (FDR; Benjamini and Hochberg, 1995). The microarray analysis was performed in biological triplicate. The raw data are available in the GEO database under accession No. GSE49919. The primers used for gene validation via RT-qPCR are provided in Supplemental Table S2.

### Ploidy analysis

The root tips (∼1 cm in length) of seedlings grown vertically on basal media for 12 d were excised and placed into a 55-mm plastic petri dish (Partec code 04-2005). After adding 500 *µ*l of extraction buffer (Partec CyStain PI absolute P; Partec GMBH, Münster, Germany), the root tips were chopped manually with a sharp razor blade for 30–60 s, incubated at room temperature for 1 min, and then passed through a Partec 50-*µ*M CellTrics disposable filter. The resulting flow-through was collected in a test tube, after which 2.0 mL of Partec staining solution, 12 *µ*L of propidium iodide, and 6 *µ*L of RNAse stock solution was added to the tube. The mixture was then incubated for 30–60 min in the dark. Subsequently, the samples were subjected to ploidy analysis via a flow cytometer (Beckman Coulter Epics Elite ESP, High-Performance Cell Sorter) under a 610-nm light channel.

### EdU staining

Five to ten seedlings were grown vertically on basal media for 8 d and then cultured for 1 h in the presence of 20 *µ*M EdU (Invitrogen) in a 12-cell plate containing liquid basal medium. The liquid medium was removed, after which the seedlings were washed twice with ddH_2_O and fixed with 4% (v/v) formaldehyde in phosphate-buffered saline (PBS) solution (pH 7.4) with 0.1% (v/v) Triton X-100 for 15 min. After fixation, the seedlings were washed with PBS three times for 5 min each. The root tissues were subsequently incubated at room temperature for 30 min in an EdU detection cocktail (Invitrogen). After three washes with PBS, the root tissues were visualized by confocal microscopy (LSM 510 META, Zeiss).

### Ribosome subunit analysis

Ribosomal and polysomal RNA were isolated as described previously (Liu et al., 2012). Briefly, 0.3 g of frozen tissue powder were extracted with 900 *μ*L of polysome extraction buffer (200 mM Tris–HCl [pH 8.5], 50 mM KCl, 25 mM MgCl_2_, 100 *μ*g mL^−1^ heparin, 50 *μ*g mL^−1^ CHX, 400 U mL^−1^ RNasin (Promega, Madison, WI, USA), 2% (v/v) polyoxyethylene 10-tridecyl ether, and 1% (v/v) deoxycholic acid). The mixture was then incubated on ice for 5 min, followed by centrifugation at 15,000 *g* for 5 min at 4°C. Then, 400 *μ*L of the supernatant was loaded onto a 10-mL continuous sucrose gradient (15%–50%) prepared with a gradient maker (ISCO, Lincoln, NE, USA), which was then centrifuged at 210,000 *g* for 3.5 h at 4°C. The distribution of the nucleic acids was examined via the UV254 absorbance profile (Brandel BR-188, Gaithersburg, MD, USA).

### Northern blot, RT-qPCR, and cRT-PCR

Total RNA was extracted from the seedlings or root tissues of wild-type (Col-0), pCOM1, and *sahy1* plants using an RNeasy Plant Mini Kit (Qiagen, Germany). For Northern blot analysis, 10 *µ*g of total RNA was separated on a 1.2% agarose/formaldehyde gel; the gel was then blotted onto a nylon membrane, which was subsequently hybridized with digoxigenin (DIG)-labeled RNA probes. The sequences of the probes used were described previously (Zhu et al., 2016) and are listed in Supplemental Table S2, and the procedure was performed according to the manufacturer’s protocol (DIG Northern Starter Kit, Roche). For RT-qPCR, the total RNA was treated with DNase I (Qiagen), after which 2 *µ*g of total RNA was reverse-transcribed with 0.5 *µ*g of an oligo dT primer using Superscript III Reverse Transcriptase (Invitrogen). qPCR was conducted with Power SYBR Green PCR Master Mix (Applied Biosystems) in an Applied Biosystems 7500 Real-Time PCR System. All primers used (Supplemental Table S2) were designed using Primer Express v.2.0 software (Applied Biosystems). The relative transcript levels were determined by the comparative threshold (CT) cycle method with *AtUBQ10* or *PP2A* (At1g13320) as endogenous controls. For all experiments, three technical replicates and three biological replicates were included. For cRT-PCR, poly(A) RNA was obtained using a Dynabead mRNA purification kit (Thermo Fisher). Total RNA and poly(A) RNA were subsequently circularized by T4 RNA ligase. First-strand cDNA was synthesized using 18S, 5.8S, or 27S-cRT primers in conjunction with Superscript III Reverse Transcriptase. After PCR amplification and gel electrophoresis, the obtained bands were cloned into a pGEM-T Easy Vector, and multiple clones were sequenced as described previously (Abbasi et al., 2010).

### Co-IP and immunoblot analyses

For total protein extraction, ∼1 g of tissue from seedlings grown on basal media for 9 d was ground in liquid N_2_ to a fine powder, which was then resuspended in extraction buffer (50 mM Tris–HCl, pH 7.5; 150 mM NaCl; 1% (v/v) Triton X-100; cOmplete EDTA-free protease inhibitor (Roche)). The lysate was centrifuged at 13,000 *g* at 4°C for 10 min. The supernatants were then transferred to fresh 1.5-mL tubes and treated with 100 *μ*L of μMACS anti-Myc microbeads (Miltteenyi Biotech Inc. CA, USA) to magnetically label the SAHY1-Myc protein, followed by incubation on ice for 30 min. The labeled supernatants were then transferred to a prewashed μMACS column placed in a separator. After three washes with washing buffer (50 mM Tris–HCl, pH 7.5; 150 mM NaCl; and 0.1% (v/v) Triton X-100), 20 *μ*L of preheated 95°C hot elution buffer (50 mM Tris–HCl, pH 6.8; 1 mM ethylenediaminetetraacetic acid (EDTA); and 1% (w/v) sodium dodycyl sulfate (SDS) was added to the column, followed by incubation for 5 min at room temperature. Subsequently, 100 *μ*L of preheated 95°C hot elution buffer was applied to the column and the eluate was collected as the immunoprecipitate. For Immunoblot analyses, the protocol was performed as described in a previous study (Cheng et al., 1996); briefly, 10 *μ*L of the eluate was separated on SDS–polyacrylamide gel electrophoresis (PAGE) gels. After blotting, the membrane was hybridized with a monoclonal Myc antibody (Merck Millipore, clone 4A6), followed by treatment with the secondary HRP-conjugated goat anti-mouse IgG antibody (Merck, ECL). For the mass spectrometry analysis, the remainder of the eluate was subjected to a trypsin protease treatment for 12 h at 37°C, followed by liquid chromatography (LC)–MS/MS analysis. An LC-nESI-Q Exactive mass spectrometer model from Thermo Fisher Scientific coupled with an on-line nanoUHPLC (Dionex UltiMate 3000 Binary RSLCnano) was utilized for protein identification and analysis. The protein ID was determined using the Proteome Discoverer software (v2.2, Thermo Fisher Scientific) with the SEQUEST and Mascot (v2.6, Matrix Sciences) search engines. MS data were searched against the TAIR 10 protein sequence database. The peptide spectrum matches (PSMs) were validated using the Percolator node, a validator algorithm that automatically conducted a decoy database search and rescored PSMs using *q*-values and posterior error probabilities. All PSMs were filtered with a *q*-value threshold of 0.01 (1% FDR) and proteins were filtered with a high confidence threshold (0.01 *q*-value, 1% FDR). For nuclear protein extraction, ∼2 g of seedlings were used for the isolation of nuclei. The procedures essentially followed the protocol described in a previous study (Fiil et al., 2008). The procedures for the Co-IP and mass spectrometry analyses of the nuclear proteins were the same as those used for total proteins.

### Accession numbers

The raw data of microarray are available in the GEO database under accession No. GSE49919. The accession numbers of genes or proteins described in this study can be found in Table 1, Supplemental Figure S2, and Table S1.

## Supplemental Data

**Supplemental Materials and Methods**

**Supplemental Figure S1.** Altered flower organ identity and reduced pollen germination in the *sahy1* mutant

**Supplemental Figure S2.** Amino acid sequence alignment and phylogenetic tree of SAHY1.

**Supplementary Figure S3.** Transcription of 45S rDNA and ribosome subunit profiling.

**Supplemental Figure S4.** Overexpression of *SAHY1-Myc* in transgenic plants.

**Supplemental Figure S5.** Validation of GeneChip data by RT-qPCR.

**Supplemental Figure S6.** The *sahy1* mutant plants are insensitive to streptomycin and CHX treatments.

**Supplemental Table S1.** Coimmunoprecipitation of SAHY1-interacting proteins.

**Supplemental Table S2.** Primers used in this study.

## Supplementary Material

kiaa085_Supplementary_Data

## Notes

P. H., M.-C.T., and H.-L. S. performed most of the experiments. Y.-H.C., Y.-Y.W., S.-G.H., W.-S.K., Y.-C.C., and Q.-V.L. performed parts of the experiments, and M.-H.C. analyzed the microarray data. S.-Y.L. and G.-Y.J. provided technical assistance and analyzed the ribosome profile. W.-H.C. conceived the original research plans, supervised the experiments, and wrote the article with help from all authors.

The author responsible for distribution of materials integral to the findings presented in this article in accordance with the policy described in the Instructions for Authors (https://academic.oup.com/plphys) is: Wan-Hsing Cheng (whcheng@gate.sinica.edu.tw).
