# Supplementary material for: The nucleolar protein SAHY1 is involved in pre-rRNA processing and normal plant growth
Source: Plant Physiol. 2020 Dec 29;185(3):1039–58. doi: 10.1093/plphys/kiaa085 (PMC8133687; doi:10.1093/plphys/kiaa085)
Supplement: kiaa085_Supplementary_Data [file kiaa085_supplementary_data.zip › pp.01338.2020-s01.pdf]

Supplemental data

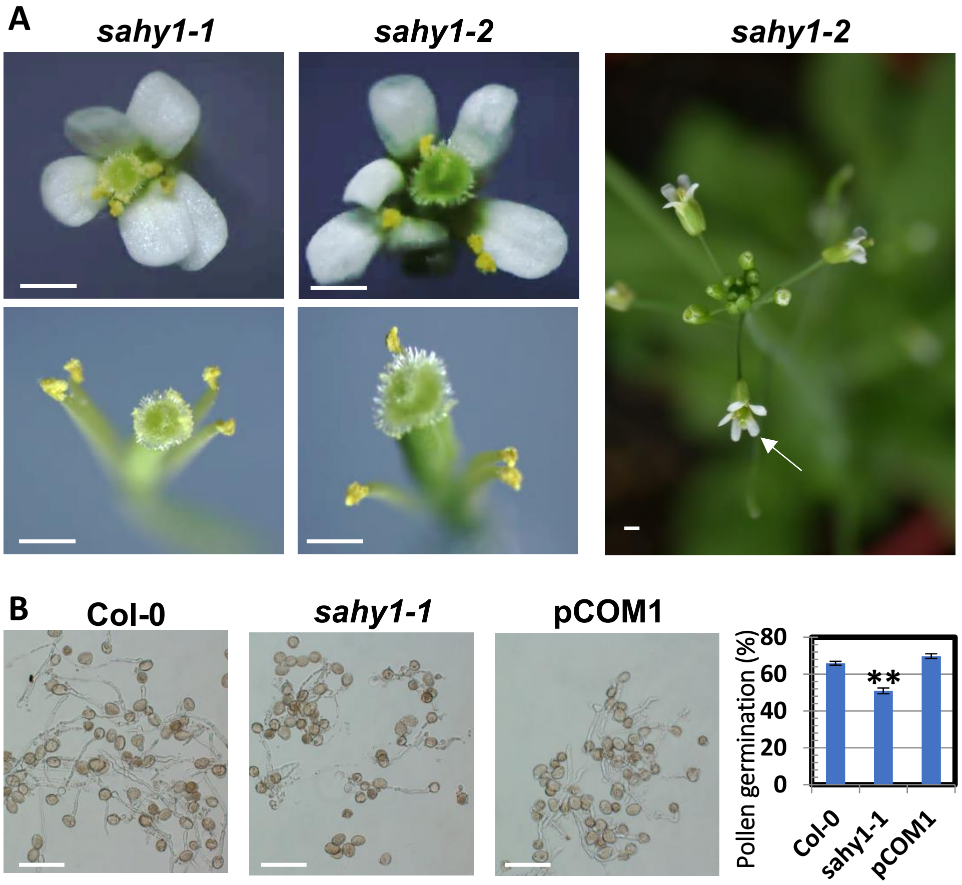

Supplemental Figure S1. Altered flower organ identity and reduced pollen germination in the *sahy1* mutant.

A, Defect of flower organs in *sahy1*. Plants were grown in soil for about 5 weeks. The arrow indicates a flower with five petals. Scale bars = 500 µm. B, Germination of pollen grains in vitro. The pollen grains were obtained from opening flowers. A total of 710, 1684, and 1580 pollen grains from the wild type, *sahy1-1*, and pCOM1 plants, respectively, were tested for germination according to Wang et al. (2008). Values are the means ± SD of three biological replicates, each with 200-600 pollen grains; **, P<0.01, Student's *t*-test. Scale bars = 500 µm.

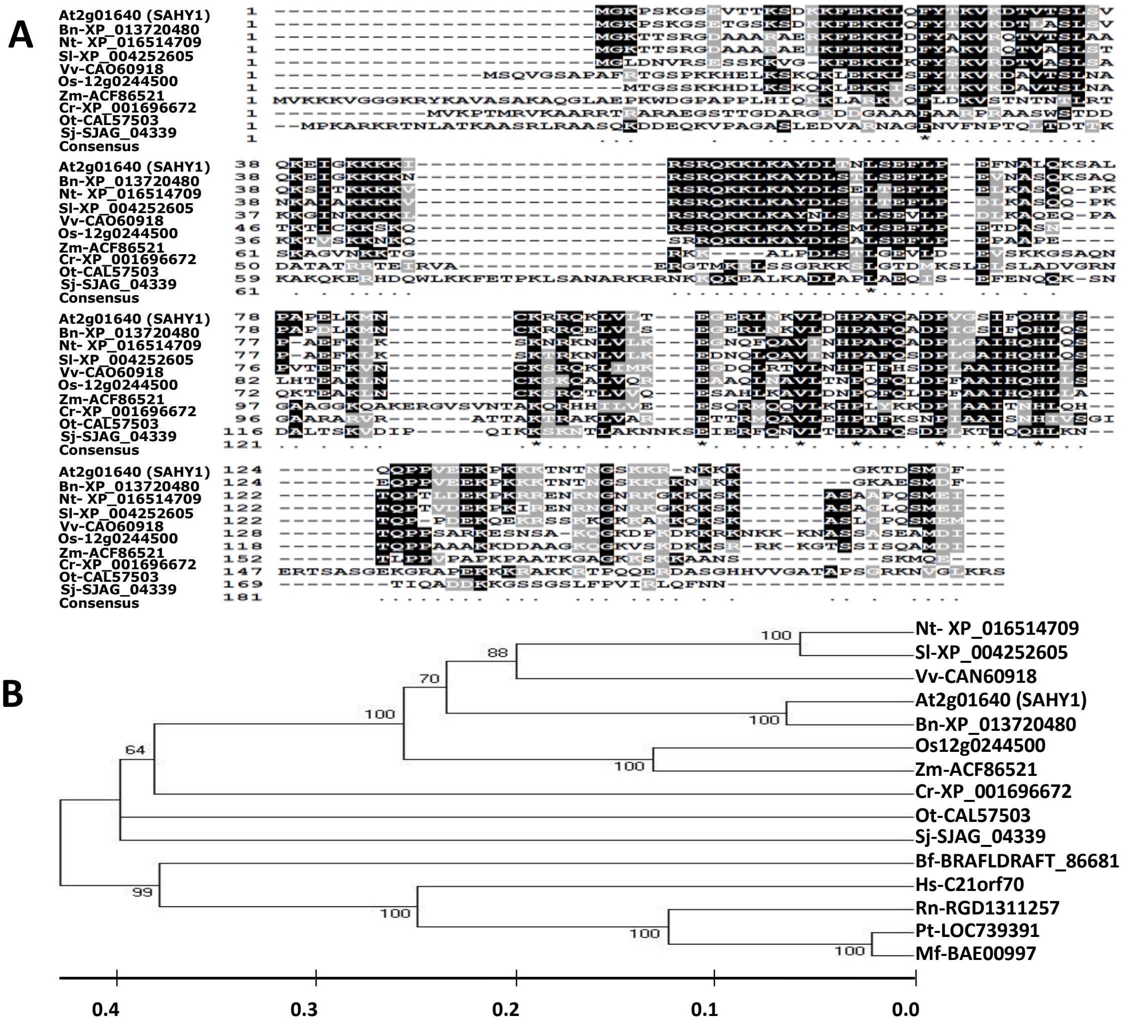

Supplemental Figure S2. Amino acid sequence alignment and phylogenetic tree of SAHY1.

A, Amino acid sequence alignment of SAHY1 and its orthologs from other species.

B, A neighbor-joining phylogenetic analysis of SAHY1 and its orthologs in plant and animal species. The scale value of 0.1 indicates 0.1 amino acid substitutions per site. The putative SAHY1 (At2G01640) homologs from other species include the following: *Brassica napus* (XP_013720480), *Nicotiana tabacum* (XP_016514709), *Solanum lycopersicum* (XP_004252605), *Oryza sativa* Japonica Group (Os12g0244500), *Zea mays* (ACF86521), *Vitis vinifera* (CAN60918), *Chlamydomonas reinhardtii* (XP_001696672), *Schizosaccharomyces japonicus* yFS275 (SJAG_04339), *Ostreococcus tauri* (CAL57503), *Branchiostoma floridae* (BRAFLDRAFT_86681), *Homo sapiens* (C21orf70), *Pan troglodytes* (LOC739391), *Rattus norvegicus* (RGD1311257) , and *Macaca fascicularis* (BAE00997).

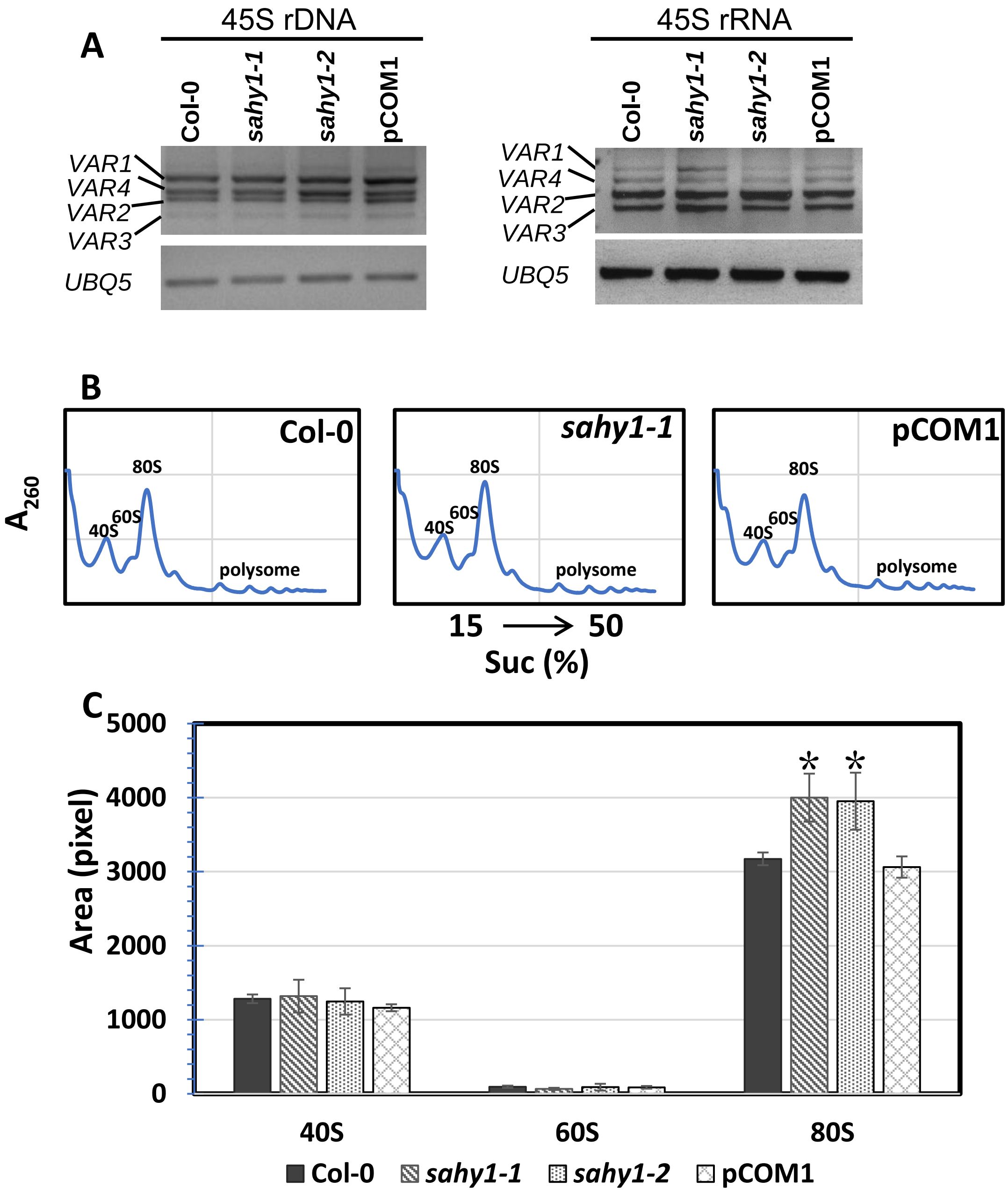

Supplemental Figure S3. Transcription of 45S rDNA and ribosome subunit profiling.

A, Expression of 45S rDNA. Genomic DNA (left panel) and cDNA (right panel) were used as templates for RT-PCR. The primers used in this study were followed by Micol-Ponce et al. (2018) and their sequences are listed in Supplemental Table S2. Three independent experiments were performed and produced consistent results.

B and C, Ribosome subunit profiling and quantification. The plants were grown vertically on basal media for 12 days and then harvested for Northern blot and ribosome subunit analyses. The values in (C) are the means ± SD of three independent experiments. *, P<0.05, Student's t-test.

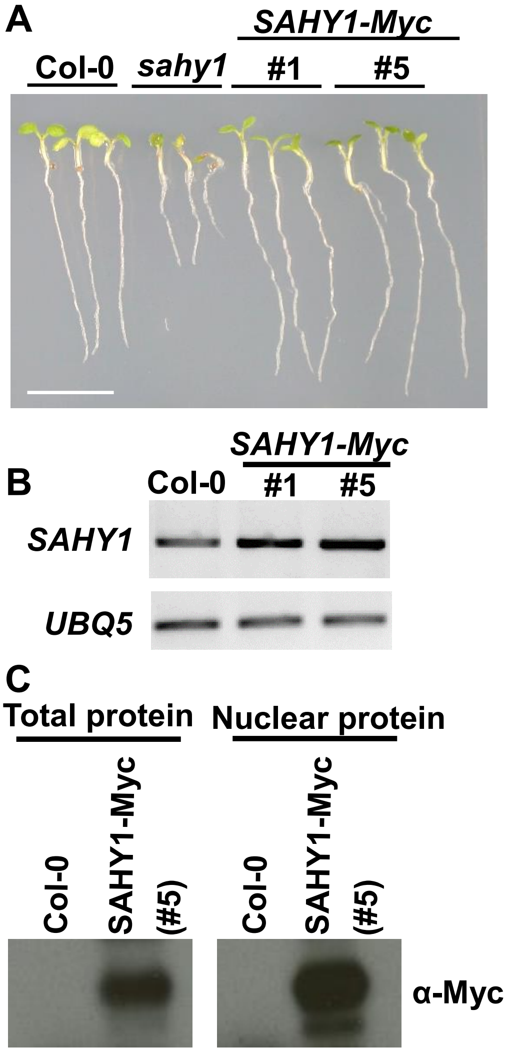

Supplemental Figure S4. Overexpression of *SAHY1-Myc* in transgenic plants.

A, Transgenic plants overexpressing *35S::SAHY1-Myc*. Seedlings were grown on basal agar plates for seven days. Scale bar = 1 cm.

B, RT-PCR showing overexpression of *SAHY1-Myc* transcripts.

C, Western blot analysis. Seedlings growing on basal agar plates for nine days were used for total and nuclear protein extractions. Each lane contained 10 μL of the eluate.

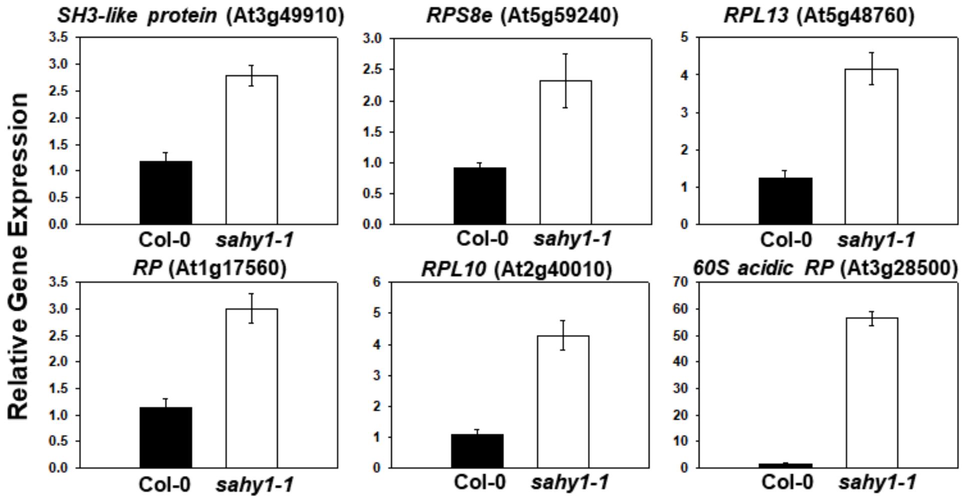

Supplemental Figure S5. Validation of GeneChip data by RT-qPCR. Genes are derived from those presented in Table 1. Values indicate the means ± SD of three biological replicates, each with three technical repeats.

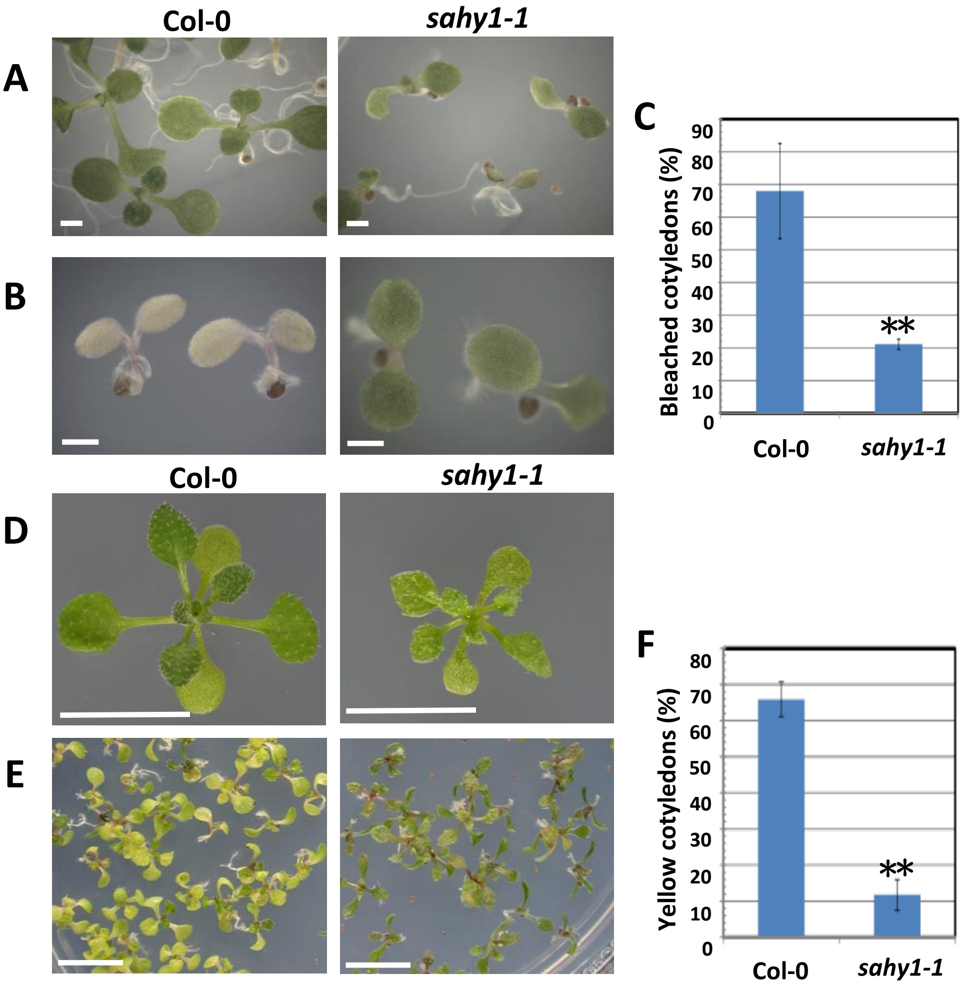

Supplemental Figure S6. The *sahy1* mutant plants are insensitive to streptomycin and CHX treatments.

A and B, Seedlings were grown on basal medium (A) or on medium supplemented with 50 μg/mL streptomycin (B) for 9 days. Scale bars = 1 mm.

C, The bleached seedlings derived from (B) were quantified and are shown in (C). Values are the means ± SD of three biological replicates, each with 50 to 100 seedlings; **, $P<0.01$, Student's *t*-test.

D and E, Seedlings were grown on basal medium (D) or medium supplemented with CHX (3.6 μM) (E) for 16 days. Scale bars = 1 cm.

F, The seedlings with chlorotic leaves derived from (E) were quantified (by normalization to total seedlings) and are shown in (F). Values are the means ± SD of three biological replicates, each with 50 to 100 seedlings; *, $P<0.01$, Student's *t*-test.

Supplemental Table S1. Co-immunoprecipitation of SAHY1 interacting proteins

Accession	Description	Extraction Method[a]	Coverage [%]	# Peptides	# PSMs	# Unique Peptides	Score Mascot	# AAs	Subcellular Locolization[b]
AT2G01640	SAHY1; Ribosome biogenesis protein	TP	43	3	37	3	461	303	nucleolus, preribosome
		NP	28	2	3	2	23		
AT1G14320.1	RPL10, Ribosome protein L10	TP	15	3	14	3	117	220	cytosol, nucleus, nucleolus
		NP	4	1	2	1	32		
AT1G18540.1	RPL6 family protein	NP	9	2	4	2	44	233	cytosol, nucleus
AT1G74060.1	RPL6 family protein	TP	12	3	7	3	50	233	cytosol, nucleolus
		NP	5	1	4	1	43		
AT2G18020.1	RPL2 family	TP	9	2	4	2	21	258	cytosol, nucleolus
		NP	10	3	6	3	82		
AT2G37190.1	RPL11 family protein	TP	22	3	20	3	140	166	cytosol, nucleus, nucleolus
		NP	9	1	4	1	33		
AT2G19730.1	Ribosomal L28e protein family	TP	13	1	2	1	51	143	cytosol, nucleus, nucleolus
AT3G49910.1	Translation protein SH3-like family protein, RPL26A	TP	15	2	4	2	36	146	cytosol, nucleus, nucleolus
		NP	15	2	7	2	33		
AT1G22780.1	RPS18A	TP	21	2	9	2	20	152	cytosol, nucleus
		NP	6	1	4	1	62		
AT3G02080.1	RPS19e family protein	TP	6	1	4	1	46	143	cytosol, nucleus
		NP	6	1	4	1	39		
AT2G1736.1	RPS4A family protein	TP	22	6	44	6	308	261	cytosol, cytosolic SSU
AT5G58420.1	RPS4A family protein	TP	6	2	5	2	40	262	cytosol, nucleolus
AT1G56070.1	LOS1, Low expression of osmotically responsive genes 1	TP	12	10	137	10	333	843	cytosol, nucleus, nucleolus, ribonucleoprotein complex
		NP	2	2	3	2	25		
AT1G01320.1	REC1, Reduced chloroplast coverage	TP	2	2	8	2	179	1797	cytosol, nucleus
		NP	1	2	3	2	30		
AT2G21660.1	GRP7, Glycine-rich RNA binding protein 7	TP	15	2	12	2	34	176	cytosol, nucleus,
		NP	6	1	2	1	51		
AT1G48920.1	Nucleolin like 1	TP	13	6	46	6	217	557	nucleus, nucleolus
AT5G44120.3	CRU1, Cruciferina	NP	8	4	9	4	193	472	nucleus
AT4G24190.1	HSP90.7, Heat shock protein 90.7	NP	1	1	2	1	72	823	cytosol, nucleus, nucleolus
AT3G18165.1	MOS4, Modifier of SNC1, 4	NP	4	1	2	1	30	253	cytosol, nucleus, nucleolus
AT5G15550.1	Transducin/WD40 repeat-like superfamily protein	NP	3	1	4	1	26	355	nucleus, nucleolus, preribosome
AT3G57150.1	HOMOLOGUE OF NAP57	NP	2	1	3	1	25	565	box H/ACA snoRNP complex, cytosol, nucleus, nucleolus
AT3G44110.1	J3, DNAJ HOMOLOGUE 3	NP	5	2	6	2	36	420	cytosol, nucleolus
AT5G04280.1	RBGB3, RNA-BINDING GLYCINE-RICH PROTEIN B3	NP	3	1	2	1	30	310	nucleus, nucleolus
AT5G46070.1	GBPL3, GUANYLATE-BINDING PROTEIN-LIKE 3	NP	1	1	2	1	49	1082	nucleus, nucleolus, plastid
AT5G67630.1	P-loop containing nucleoside triphosphate hydrolases superfamily protein	NP	5	2	3	2	48	469	nucleus, nucleolus, plastid
AT5G20020.1	RAN2, RAS-RELATED GTP-BINDING NUCLEAR PROTEIN 2	NP	5	1	2	1	26	221	nucleus, nucleolus

[a]Both total proteins (TP) and nuclear proteins (NP) were extracted from seedlings grown on basal media for 9 days. [b]Subcellular localization is based on GO cellular component of TAIR Araport11. The candidates of SAHY1 interacting proteins have experimental q value < 0.01. PSM, peptide spectrum matches.

**Supplemental Table S2. Primers used in this study**		
AGI number	Description	Primer sequences (5'-->3')
At2g01640	*SAHY1*	F_CCAAGCAAAGGATCGGAAGTG
		R_CTGTTGACTCAGCAAGTGCTG
At3g62250	*UBQ5*	F_GTGGTGCTAAGAAGAGGAAGA
		R_TCAAGCTTCAACTCCTTCTTT
At3g49910	*Translation protein SH3-like family protein*	F_TGTTCAGGTTTACCGTCGCAA
		R_CGTTCACGGTTGTTCCATTCA
At5g59240	*Ribosomal protein S8e family protein*	F_GCTTTGAGGCTCGATACTGGAA
		R_CGCCACATCCAAGATCCTAGTC
At5g48760	*Ribosomal protein L13 family protein*	F_TGCGTCGATCACCGCTAAA
		R_TCTTCGCACCGGACAATGA
At1g17560	*Ribosomal protein*	F_GACTGGTACCCGAGTGTTTGGT
		R_TGTTTCCTGAGGCGCATCT
At2g40010	*Ribosomal protein L10 family protein*	F_CGCCTCACATGTTCCTCAATG
		R_GCTAAGGCAACAGCAAGAACGT
At3g28500	*60S acidic ribosomal protein family*	F_GCCGGTTGCTGAATCTAAGAA
		R_TGCCTGCATCGTCAGAAGA
AT1G13320	*PP2A*	F_TAACGTGGCCAAAATGATGC
		R_GTTCTCCACAACCGCTTGGT
p1 probe	For Northern blot	F_TCGATCACGGCAATTCCCCGCCACAT
		R_CCTCTCAAACGCAATGGAAAGAGA
p2 probe	For Northern blot	F_GATCCGGCGGGCAAGGAATCGGCTAA
		R_GAAACCGGCCCACCGAGAGTGGTG
p3 probe	For Northern blot	F_AGACTTCAGTTCGCAGCACAGCATCC
		R_GCCCACACTCCGTCTCCGGGGAGG
p4 probe	For Northern blot	F_TCCAGGCGTCCTTGGCTCGGATTTAG
		R_GCCAACCGCGTGCGGTAACACACG
p5 probe	For Northern blot	F_ACAAGAGCGACCGATAAAATGTAATGGATC
		R_TGAGCACTCTAATTTCTTCAAAGT
45S rDNA	For *VAR* expression	F_GACAGACTTGTCCAAAACGCCCACC
		R_CTGGTCGAGGAATCCTGGACGATT
r2	For cRT-PCR	GCAGACGACTTAAATACGCGAC
r3	For cRT-PCR	GTCGTTCTGTTTTGGACAGGTATCGA
r4	For cRT-PCR	CTCCGCTTATTGATATGCTTAAAC
r5	For cRT-PCR	GACTTATGGAAGGGACGCAT
r6	For cRT-PCR	GGTTTCTTAGCCGATTCCTTGC
r8	For cRT-PCR	CTTGTTACGACTTCTCCTTCCT
r9	For cRT-PCR	CCGAGATATCCGTTGCCGAGAGT
r10	For cRT-PCR	CGATCCACTGAGATTCAGCCCT

MATERIALS and METHODS

Analysis of Mature Pollen Germination

To examine pollen germination, approximately 20 opening flowers per genotype were harvested and placed in a 1.5 mL Eppendorf tube containing 800 µl of germination solution composed of 1 mM KCl, 5 mM $CaCl_2$, 0.8 mM $MgSO_4$, 1.5 mM boric acid, 10 µM myoinositol, MES (pH 5.8), and 15% sucrose (Suc). After vortexing for five min, the flower debris was removed and the supernatant was centrifuged at 11,000 rpm for 5 min. Subsequently, the supernatant was discarded, and 100 µl of germination solution with 19.8% Suc was added to resuspend the pollen grains by pipetting. Then, the suspended pollen grains were placed on a 1% agarose gel containing germination solution in 5 cm Petri dishes for overnight incubation in the dark. The procedure was essentially followed the protocol previously described (Wang et al., 2008).
