# Supplementary figures and images for: The nucleolar protein SAHY1 is involved in pre-rRNA processing and normal plant growth
Source: Plant Physiol. 2020 Dec 29;185(3):1039–58. doi: 10.1093/plphys/kiaa085 (PMC8133687; doi:10.1093/plphys/kiaa085)

## Slide 1
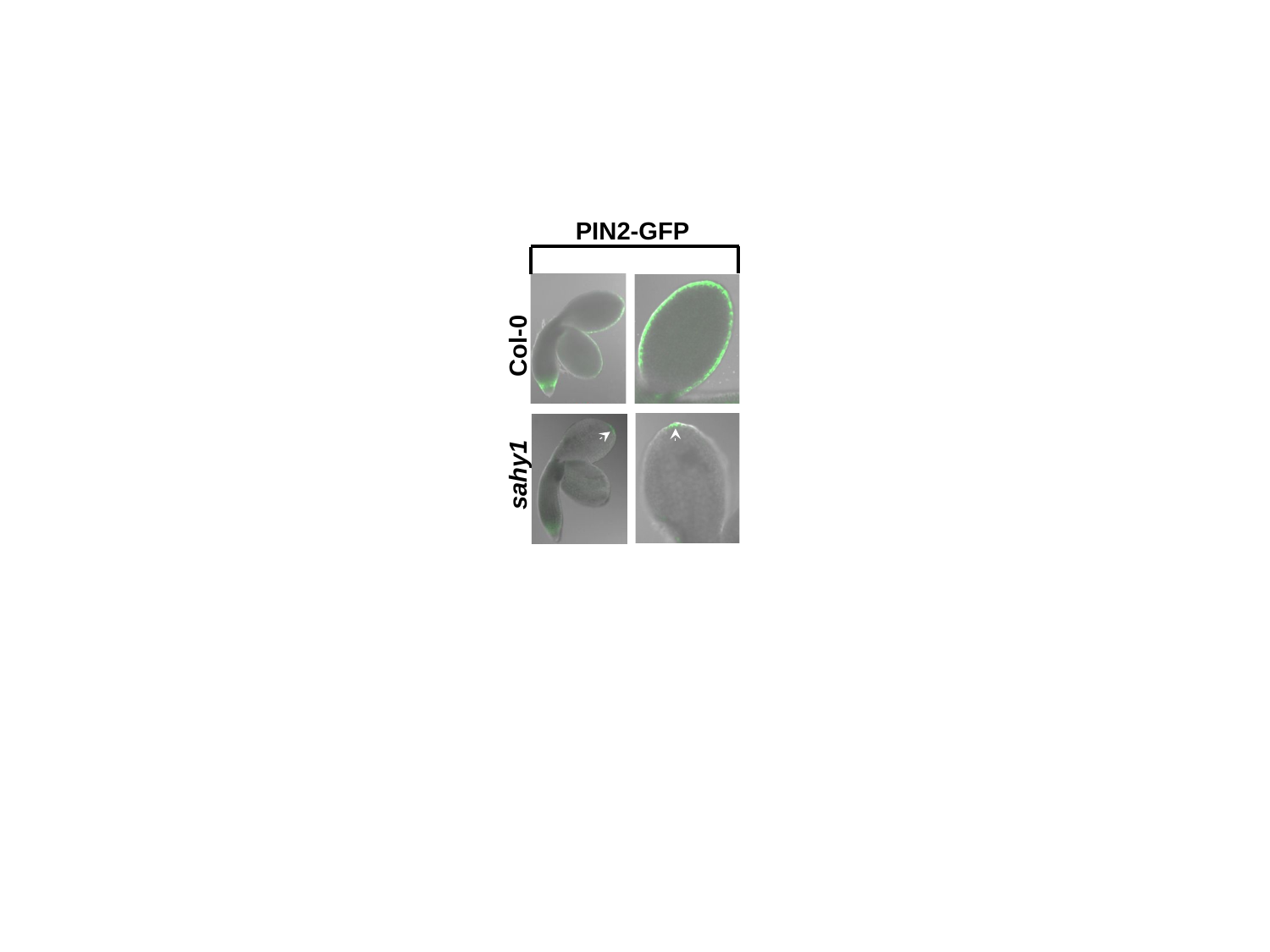

PIN2-GFP
Col-0
sahy1

Supplement: kiaa085_Supplementary_Data [file kiaa085_supplementary_data.zip › pp.01338.2020-s02.pptx]
